# Supplementary material for: Antiprotozoal Activity Against Entamoeba histolytica of Flavonoids Isolated from Lippia graveolens Kunth
Source: Molecules. 2020 May 26;25(11):2464. doi: 10.3390/molecules25112464 (PMC7321152; doi:10.3390/molecules25112464)
Supplement: Supplementary file 1 [file molecules-25-02464-s001.pdf]

Figures with supplementary spectroscopic data

Flavonoids with Antiprotozoal activity against *Entamoeba histolytica* isolated from *Lippia graveolens* Kunth (Mexican oregano)

*Ramiro Quintanilla-Licea[1], Javier Vargas-Villarreal[2], María Julia Verde-Star[1], Verónica Mayela Rivas-Galindo[3], Ángel David Torres-Hernández[1]

[1]Universidad Autónoma de Nuevo León (UANL), Facultad de Ciencias Biológicas, Av. Universidad S/N, Cd. Universitaria, San Nicolás de los Garza, C.P. 66455 Nuevo León, Mexico; [2]Centro de Investigaciones Biomédicas del Noreste (CIBIN), Laboratorio de Bioquímica y Biología Celular, Dos de abril esquina con San Luis Potosí, C.P. 64720 Monterrey, Mexico; [3]Universidad Autónoma de Nuevo León (UANL), Facultad de Medicina, Madero y Aguirre Pequeño, Mitras Centro, Monterrey, C.P. 64460 Nuevo León, Mexico

***Corresponding author**: Ramiro.quintanillalc@uanl.edu.mx

Table of Contents **Page**

S1: ^{1}H-NMR Spectrum of Pinocembrin (**1**) d_6-DMSO, 400 MHz……………………………………………….…………………3

S2: ^{1}H-NMR Spectrum (Ext. 7.5 – 2.5 ppm) of Pinocembrin (**1**) d_6-DMSO, 400 MHz……………………………….....…………4

S3: COSY Spectrum of Pinocembrin (**1**) d_6-DMSO, 400 MHz……………………………………………………………………..5

S4: ^{13}C-NMR Spectrum of Pinocembrin (**1**) (d_6-DMSO, 100 MHz)…………………………………...………………..6

S5: DEPT-135 Spectrum of Pinocembrin (**1**) (d_6-DMSO, 100 MHz)…………………………………………………...……..7

S6: HSQC Spectrum (Ext. ^{1}H: 2.0-8.2 ppm; ^{13}C: 40-170 ppm) of Pinocembrin (**1**) (d_6-DMSO, 400 MHz, 100 MHz)………………..8

S7: ^{1}H-NMR Spectrum of Sakuranetin (**2**) d_6-DMSO, 400 MHz……………………………………………………………..9

S8: ^{1}H-NMR Spectrum (Ext. 6.4 – 8.0 ppm) of Sakuranetin (**2**) d_6-DMSO, 400 MHz………………………………...……………10

S9: ^{1}H-NMR Spectrum (Ext. 2.5 – 4.0 ppm) of Sakuranetin (**2**) d_6-DMSO, 400 MHz……………………….……………………..11
S10: COSY Spectrum of Sakuranetin (**2**) d_6-DMSO, 400 MHz………………………………………………………….…….12
S11: ^{13}C-NMR Spectrum of Sakuranetin (**2**) (d_6-DMSO, 100 MHz)……………………………………………...……..…13
S12: DEPT-135 Spectrum of Sakuranetin (**2**) (d_6-DMSO, 100 MHz)…………………………………………………...……..14
S13: HSQC Spectrum (Ext. ^{1}H: 2.0-7.6 ppm; ^{13}C: 40-140 ppm) of Sakuranetin (**2**) (d_6-DMSO, 400 MHz, 100 MHz)…………….…15
S14: ^{1}H-NMR Spectrum of Cirsimaritin **(3**) d_6-DMSO, 400 MHz………………………………………………………………..16
S15: ^{1}H-NMR Spectrum (Ext. 6.8 – 8.0 ppm) of Cirsimaritin (**3**) d_6-DMSO, 400 MHz…………..……………...………………17
S16: COSY Spectrum of Cirsimaritin (**3**) d_6-DMSO, 400 MHz…………………………………………………...…..………..18
S17: COSY Spectrum (Ext ^{1}H: 5.0-9.4 ppm; ^{1}H: 5.0-9.4 ppm) of Cirsimaritin (**3**) d_6-DMSO, 400 MHz……………………………19
S18: ^{13}C-NMR Spectrum of Cirsimaritin (**3**) (d_6-DMSO, 100 MHz)……………………..…………...…………………………20
S19: HSQC Spectrum (Ext. ^{1}H: 6.2-8.4 ppm; ^{13}C: 85-145 ppm) of Cirsimaritin (**3**) (d_6-DMSO, 400 MHz, 100 MHz).......................21
S20: ^{1}H-NMR Spectrum of Naringenin (**4**) d_6-DMSO, 400 MHz……………………………….……………………………..22
S21: ^{1}H-NMR Spectrum (Ext. 8.0 – 2.0 ppm) of Naringenin (**4**) d_6-DMSO, 400 MHz………………………………………………23
S22: COSY Spectrum of Naringenin (**4**) d_6-DMSO, 400 MHz……………………………………………...………………24
S23: ^{13}C-NMR Spectrum of Naringenin (**4**) (d_6-DMSO, 100 MHz)…………………………..…………………………….…25
S24: DEPT-135 Spectrum of Naringenin (**4**) (d_6-DMSO, 100 MHz)…………………………….…………………...……..26
S25: HSQC Spectrum (Ext. ^{1}H: 0-9.0 ppm; ^{13}C: 10-180 ppm) of Naringenin (**4**) (d_6-DMSO, 400 MHz, 100 MHz)…….….………….27
S26: IR spectrum of Pinocembrin **1**……………………………………………………………………………...……..……..28
S27: IR spectrum of Sakuranetin **2**…………………………………………………………………………………………..…29
S28: IR spectrum of Cirsimaritin **3**……………………………………………………………………..…………….……..…..30
S29: Table 1. ^{1}H-NMR spectral data of compounds **1-4** (400 MHz, DMSO-d_6)…… ………………………………………..31
S30: Table 2. ^{13}C-NMR spectral data of compounds **1-4** (100 MHz, DMSO-d_6)……………… …………….32

S1: ^{1}H-NMR Spectrum of Pinocembrin (**1**) d_6-DMSO, 400 MHz

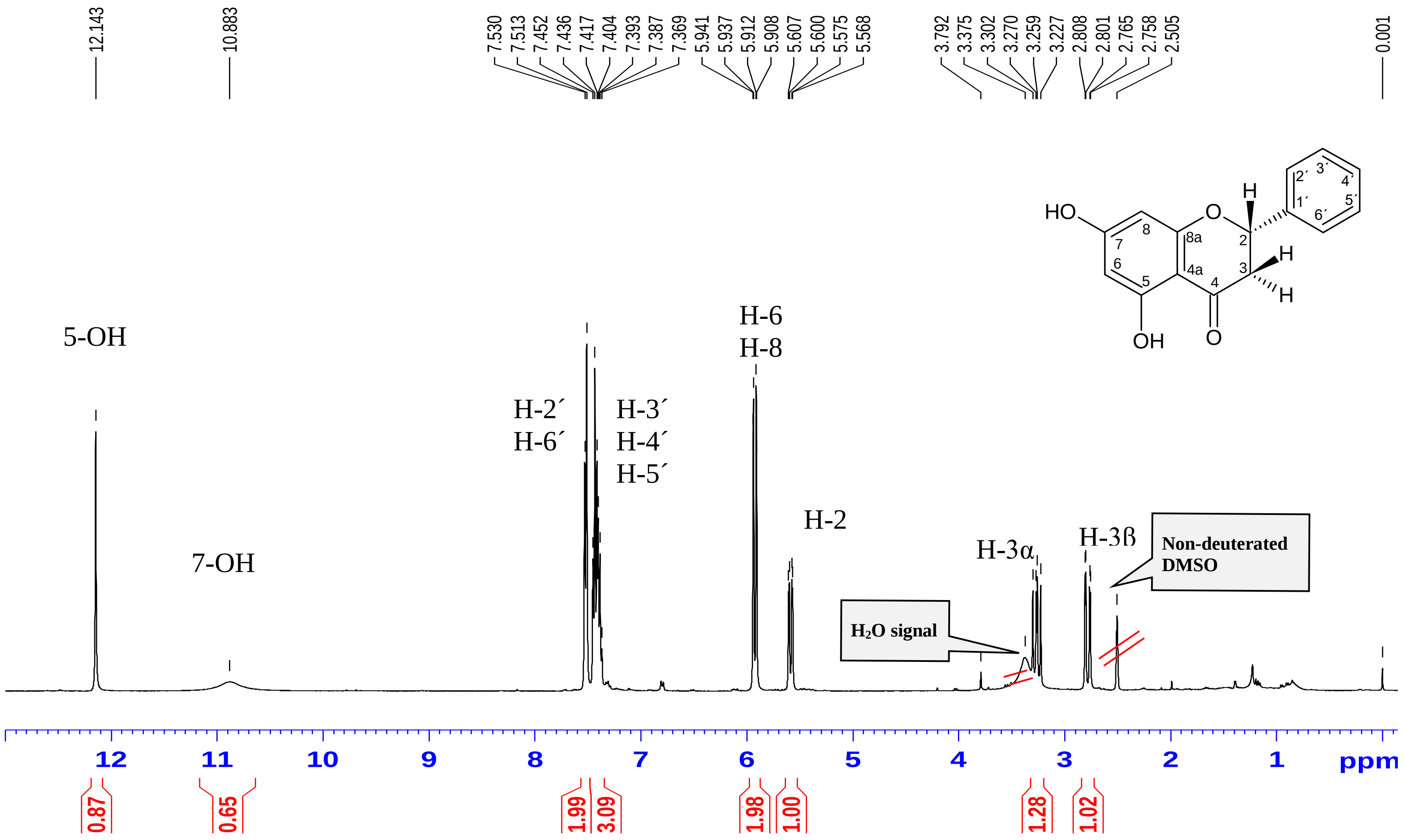

S2: ^{1}H-NMR Spectrum (Ext. 7.5 – 2.5 ppm) of Pinocembrin (**1**) d_6-DMSO, 400 MHz

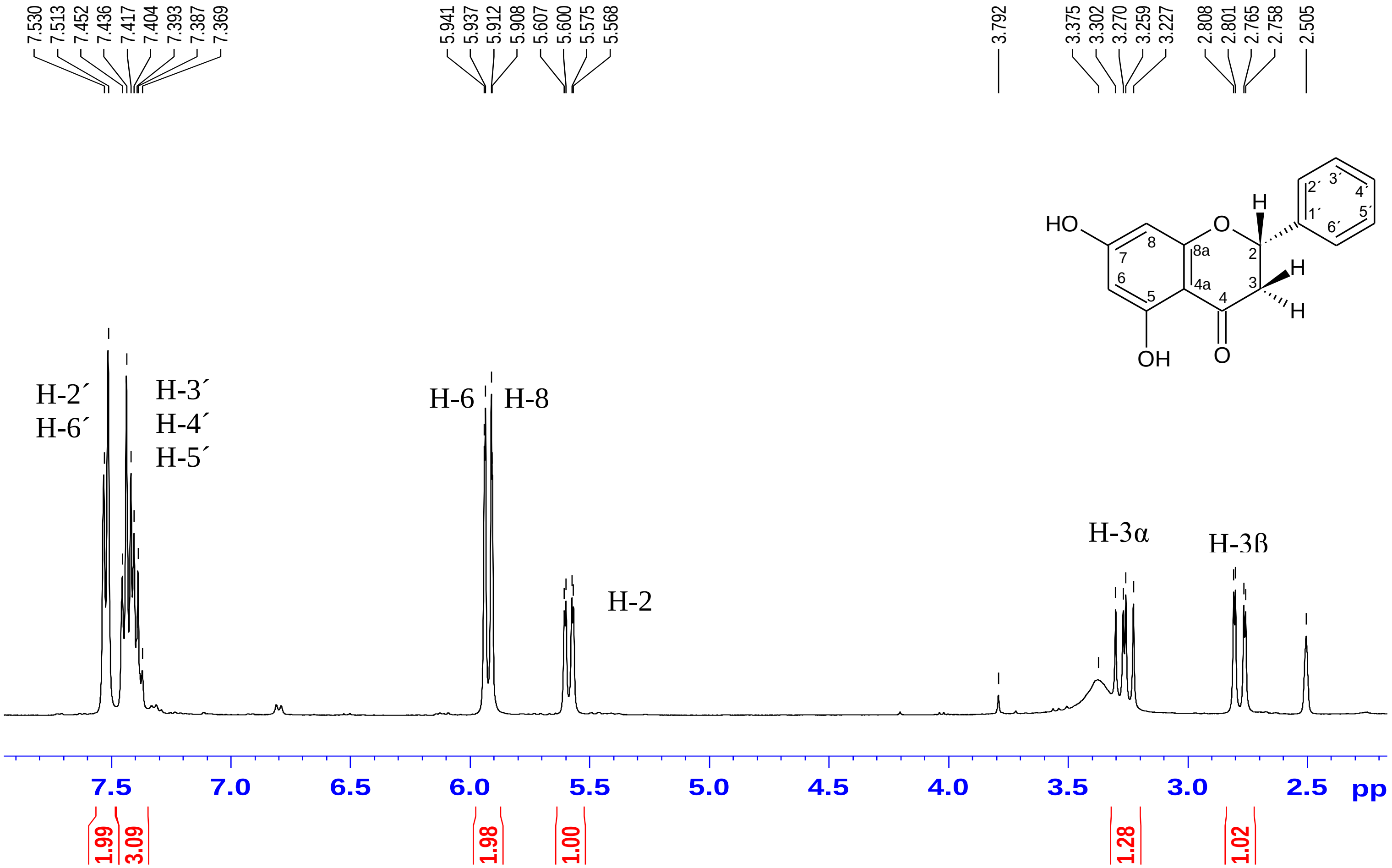

S3: COSY Spectrum of Pinocembrin (**1**) *d*$_6$-DMSO, 400 MHz

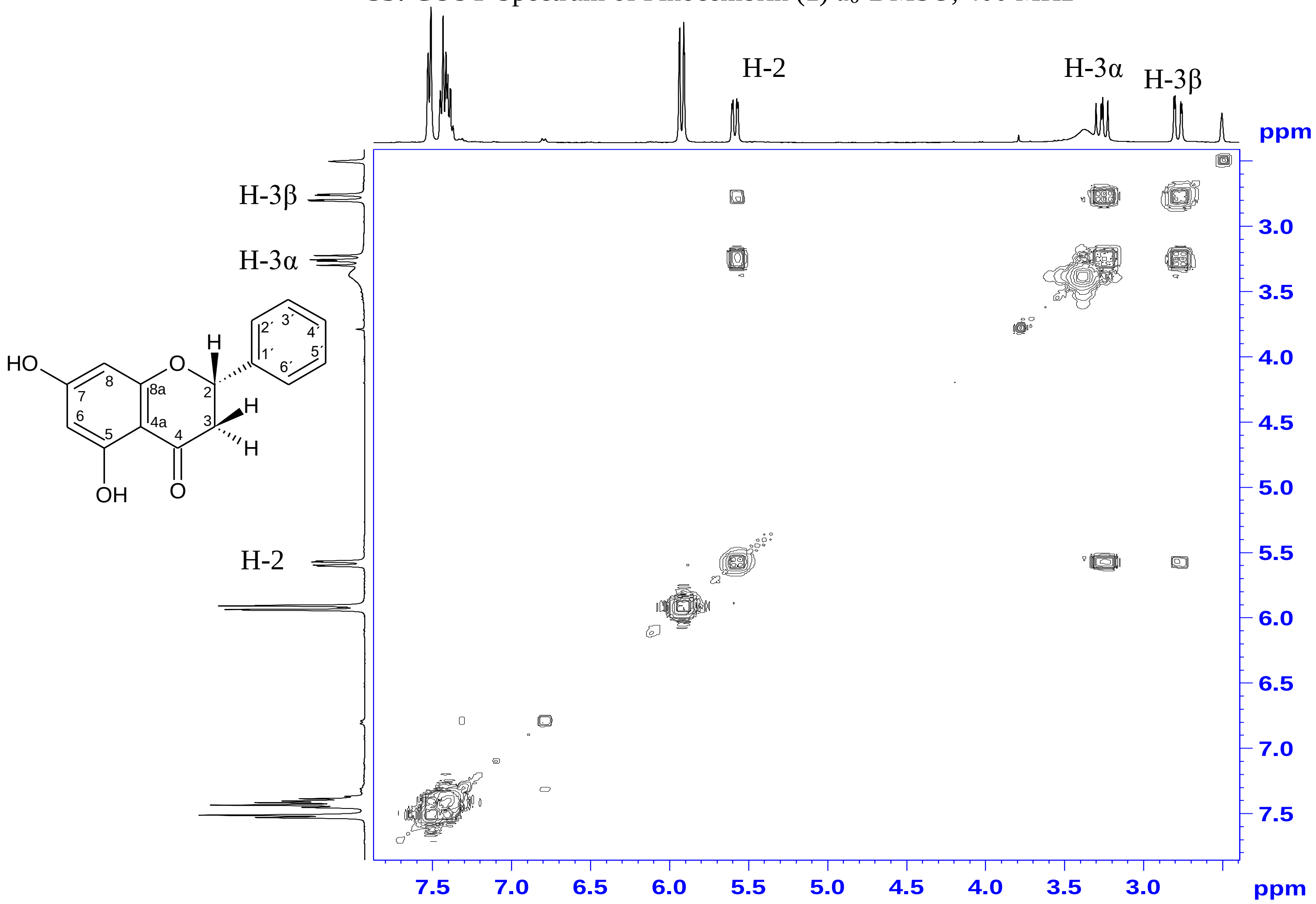

S4: ^{13}C-NMR Spectrum of Pinocembrin (**1**) (d_6-DMSO, 100 MHz)

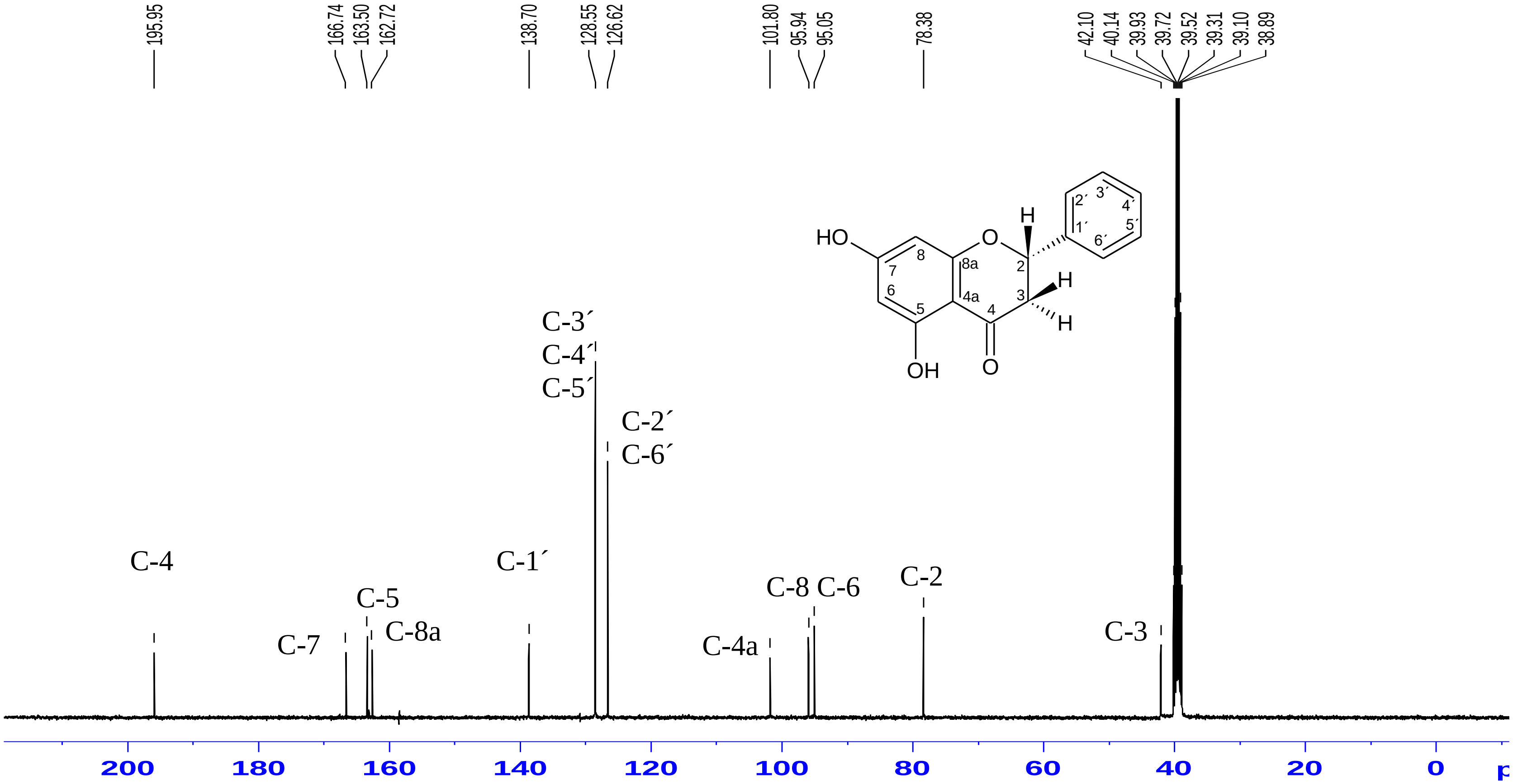

S5: DEPT-135 Spectrum of Pinocembrin (**1**) (*d_6*-DMSO, 100 MHz)

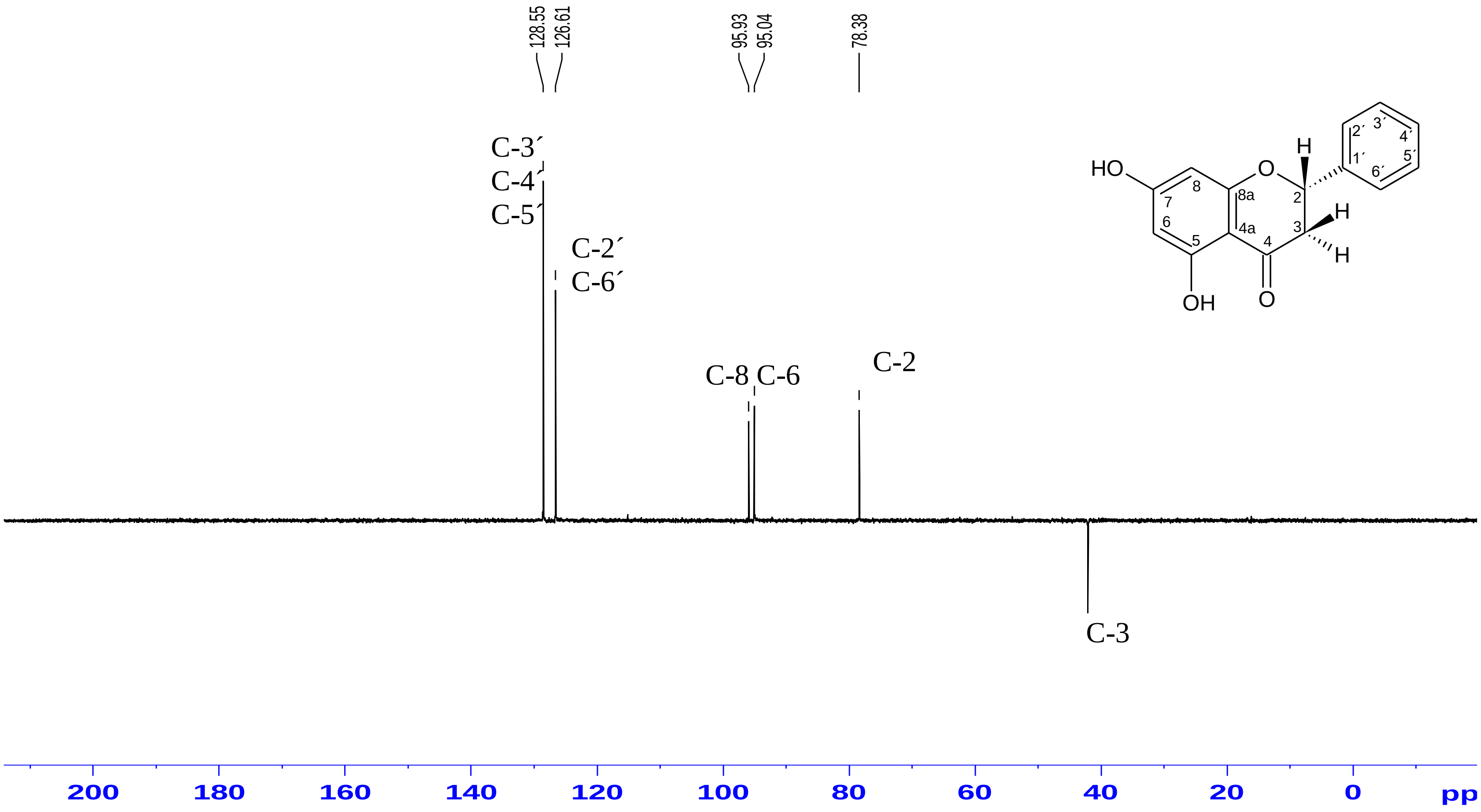

S6: HSQC Spectrum (Ext. ^{1}H: 2.0-8.2 ppm; ^{13}C: 40-170 ppm) of Pinocembrin (**1**) (d_6-DMSO, 400 MHz, 100 MHz)

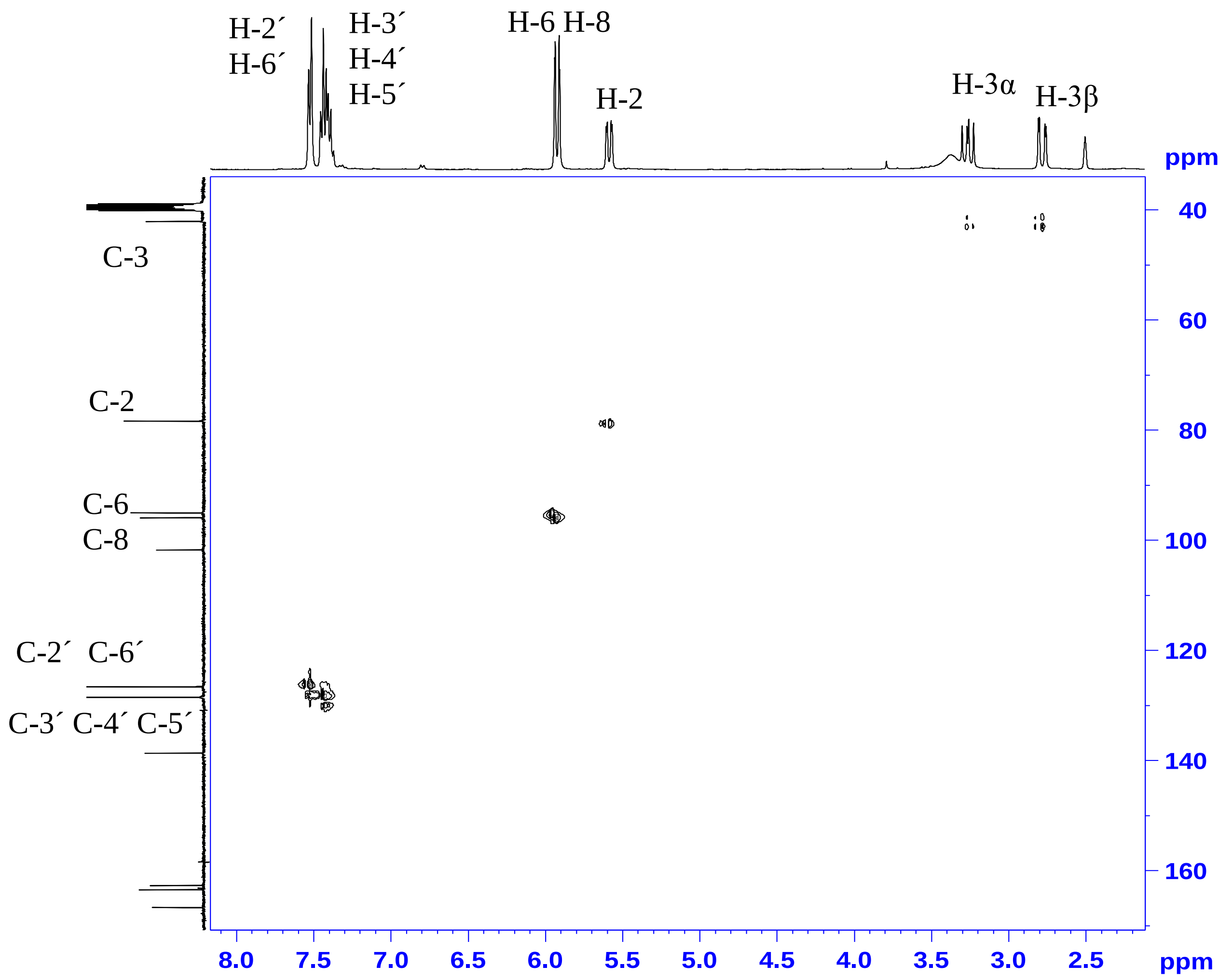

S7: ^{1}H-NMR Spectrum of Sakuranetin **(2)** d_6-DMSO, 400 MHz

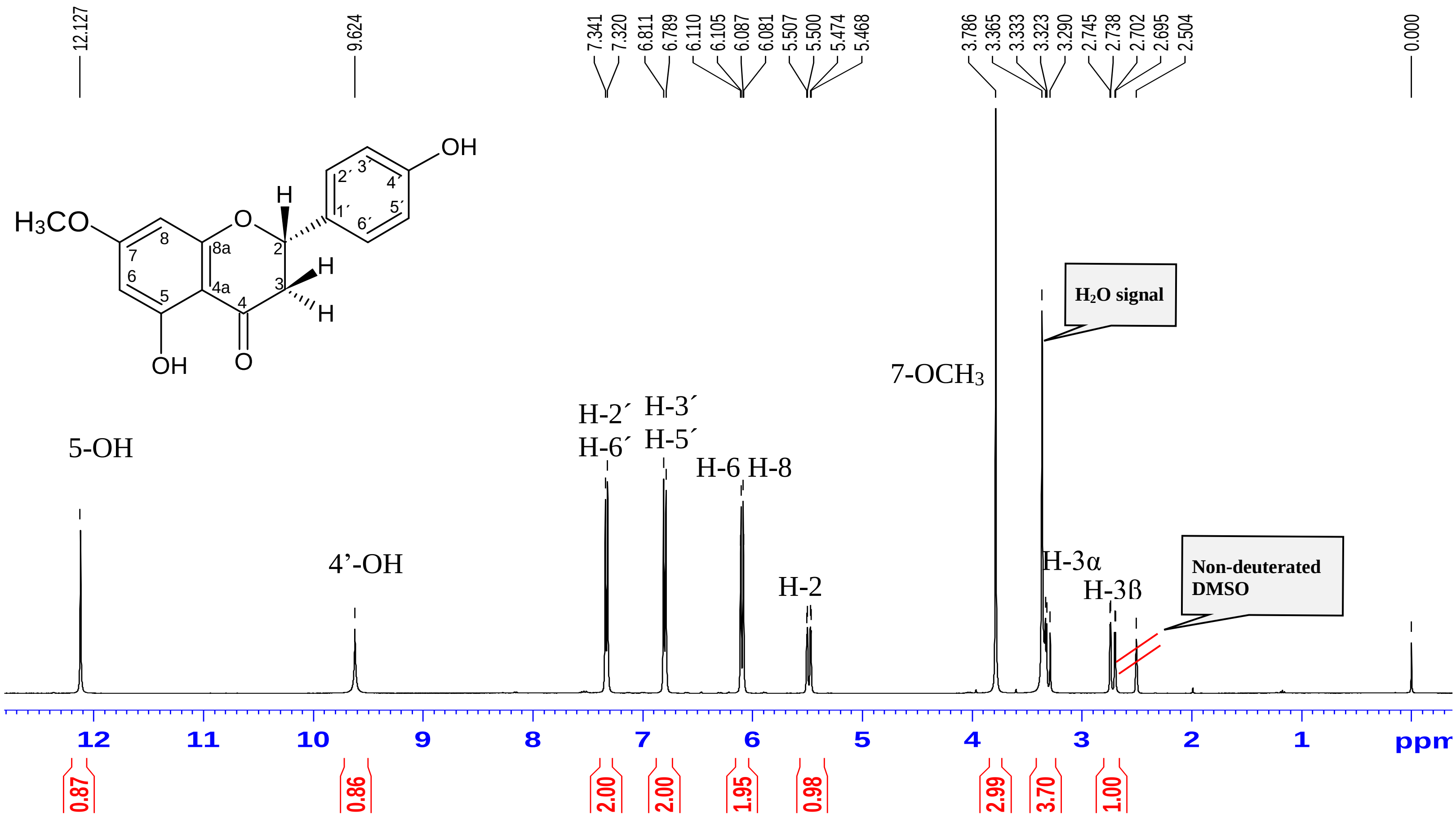

S8: ^{1}H-NMR Spectrum (Ext. 6.4 – 8.0 ppm) of Sakuranetin (**2**) *d_6*-DMSO, 400 MHz

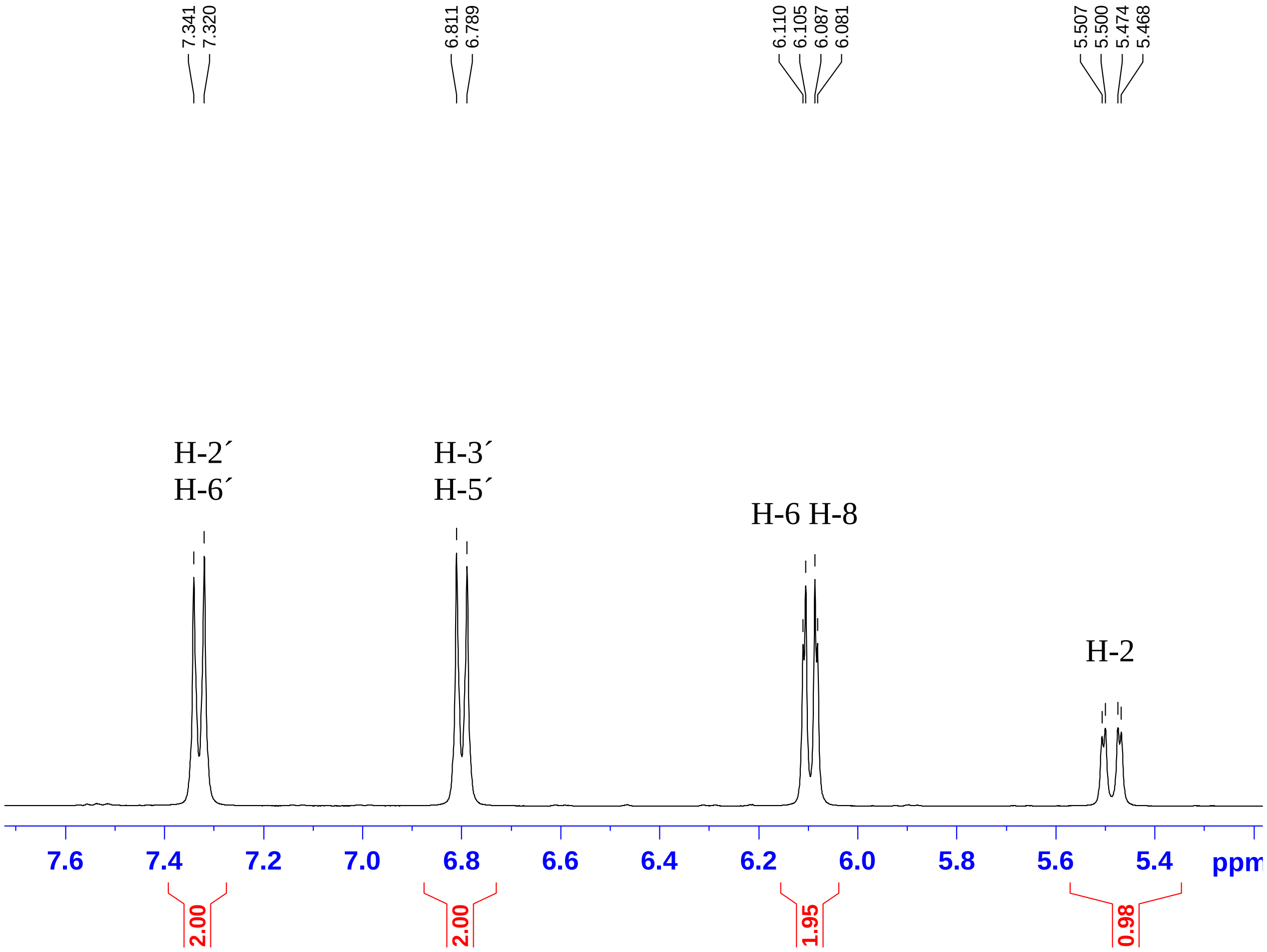

S9: ^{1}H-NMR Spectrum (Ext. 2.5 – 4.0 ppm) of Sakuranetin (**2**) d_6-DMSO, 400 MHz

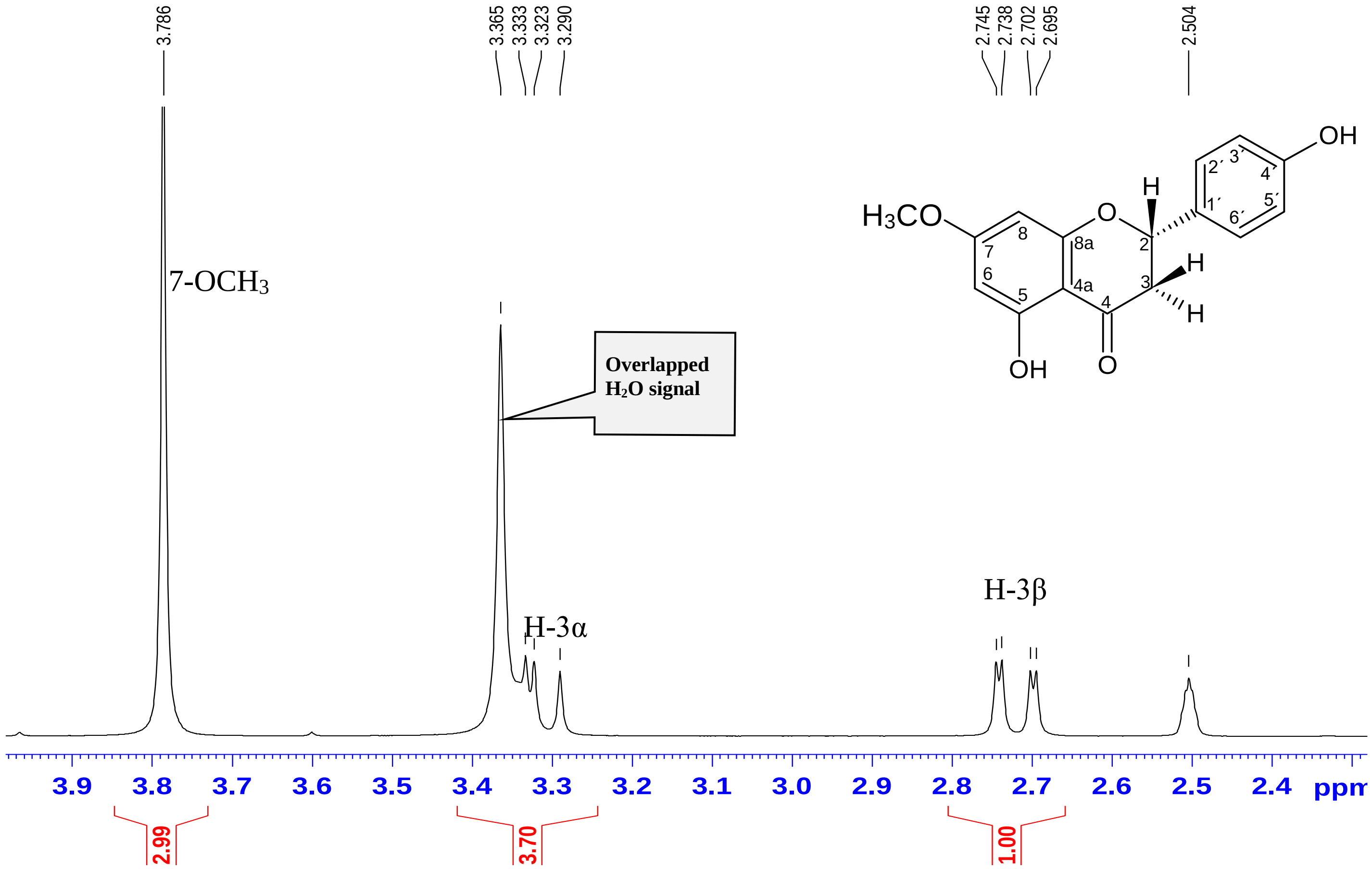

S10: COSY Spectrum of Sakuranetin (**2**) d_6-DMSO, 400 MHz

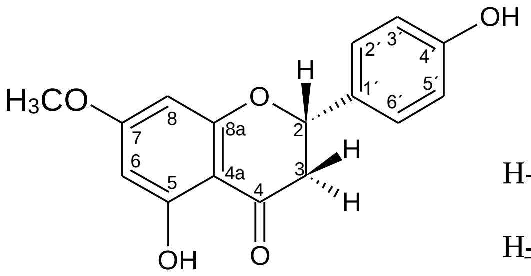

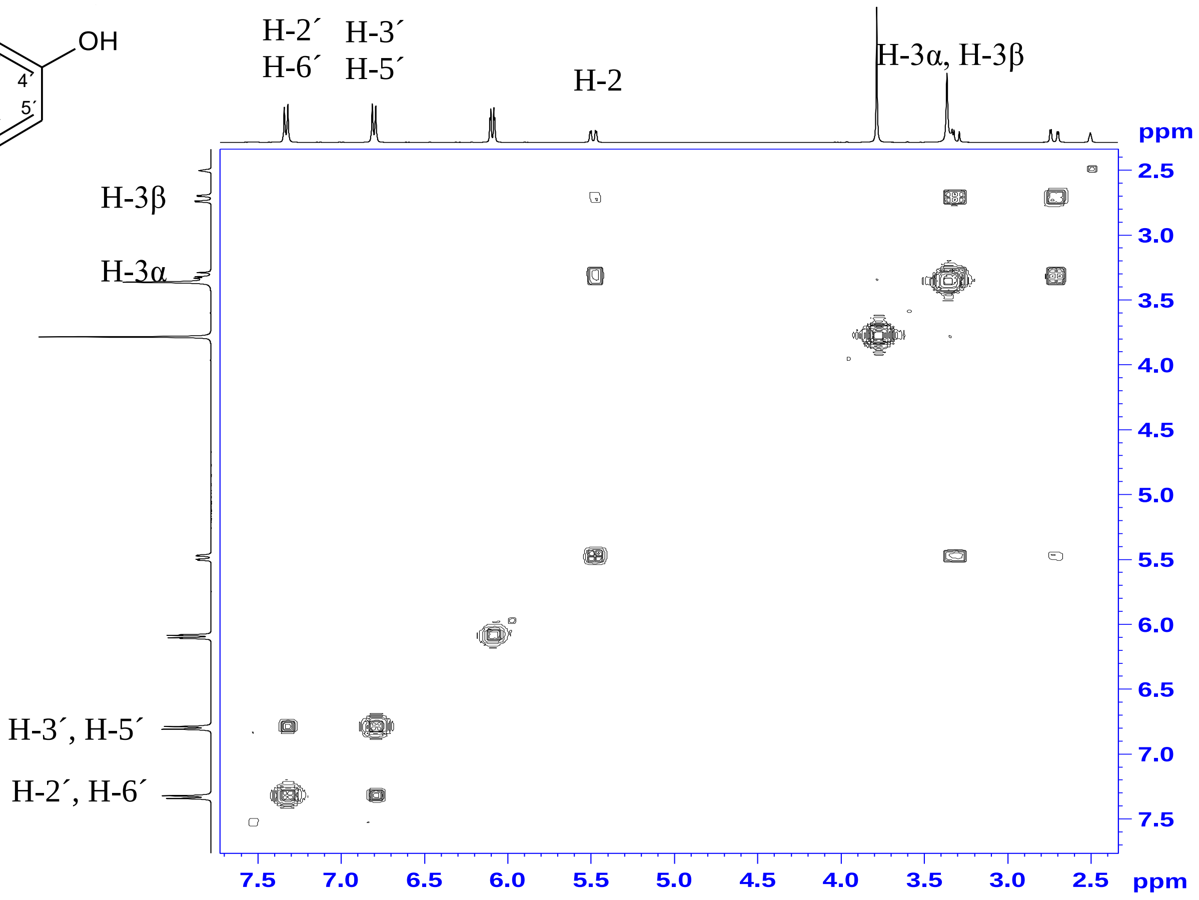

S11: ^{13}C-NMR Spectrum of Sakuranetin (**2**) (*d*$_6$-DMSO, 100 MHz)

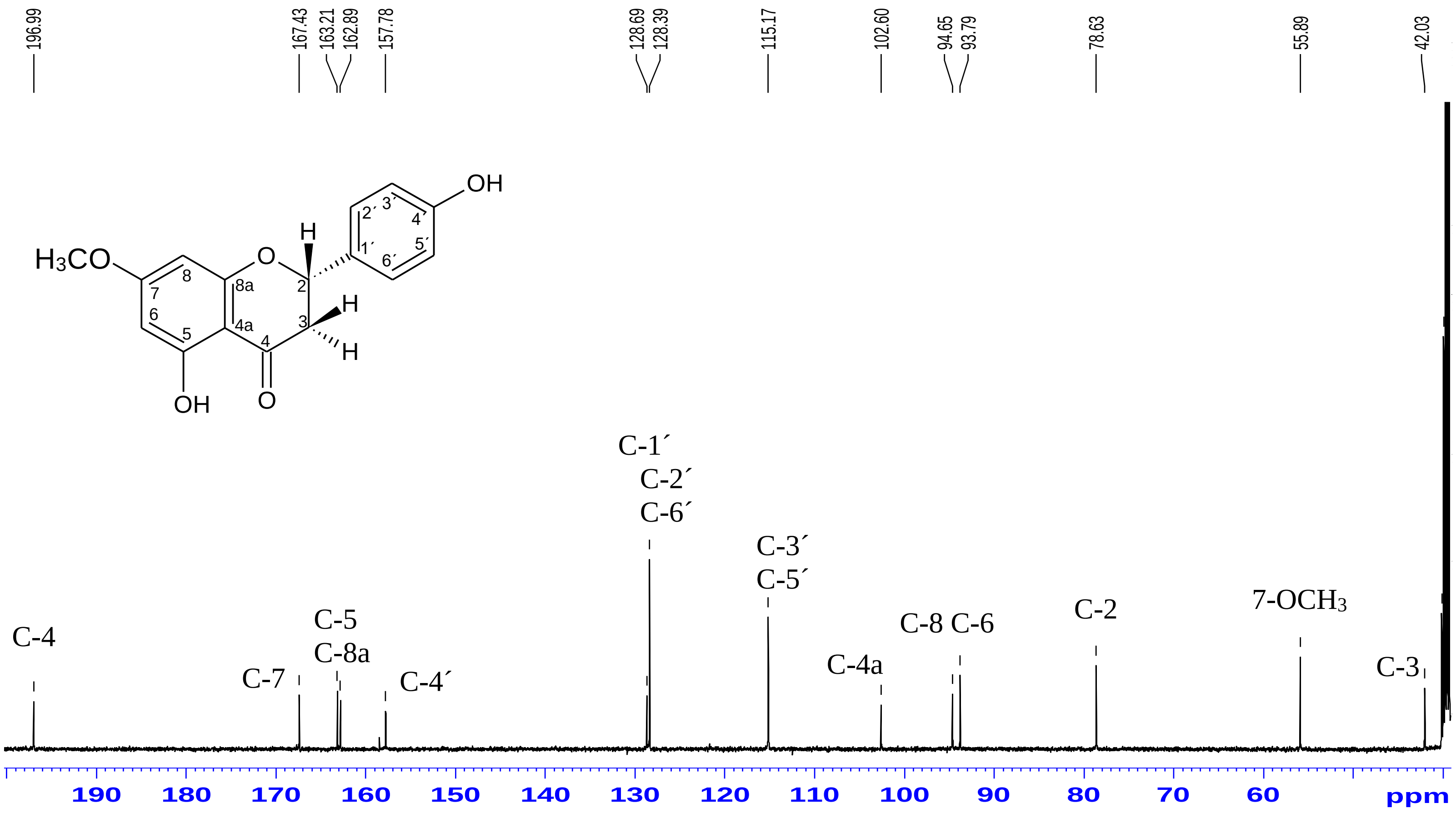

S12: DEPT-135 Spectrum of Sakuranetin (**2**) (d_6-DMSO, 100 MHz)

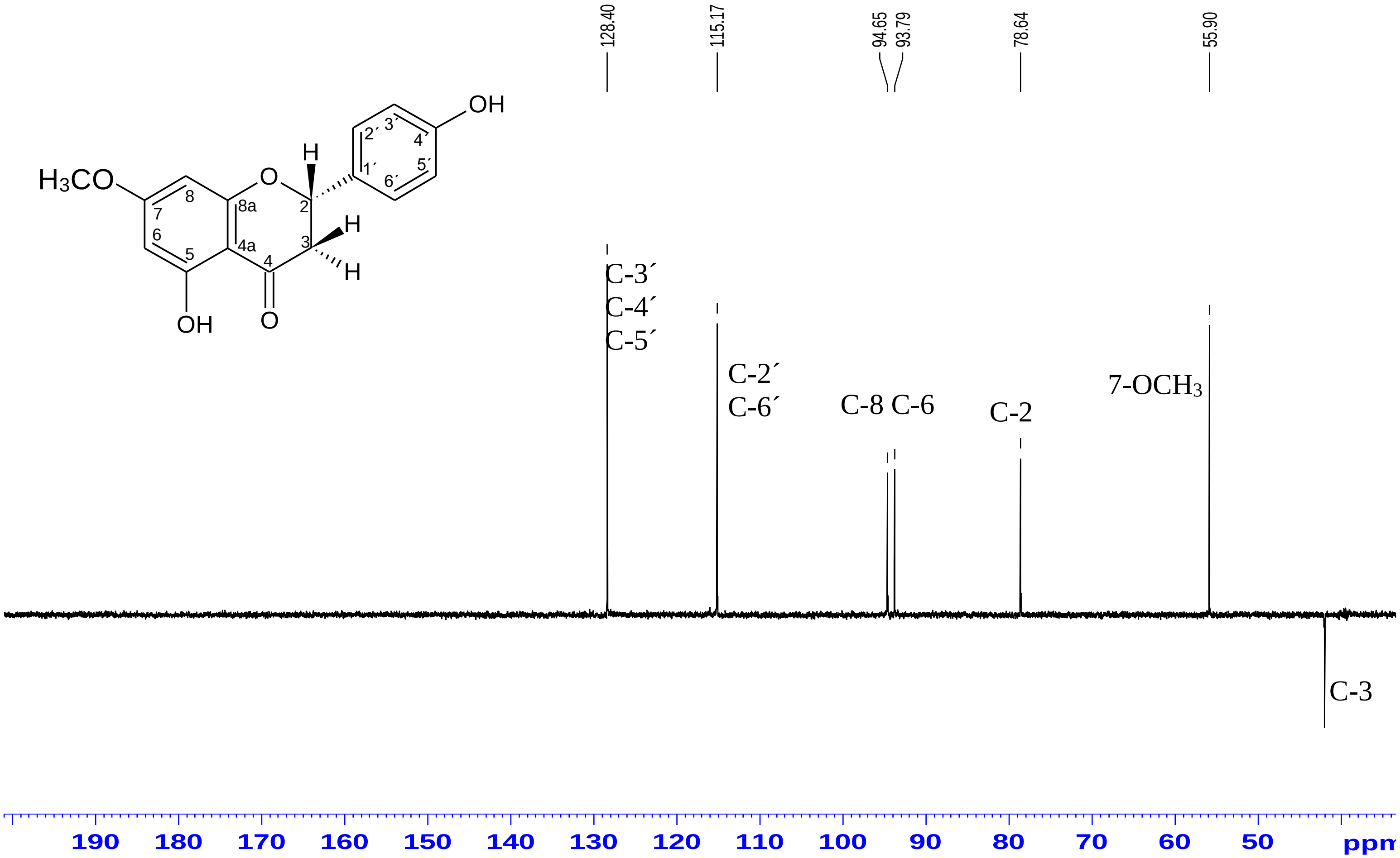

S13: HSQC Spectrum (Ext. ^{1}H: 2.0-7.6 ppm; ^{13}C: 40-140 ppm) of Sakuranetin (**2**) (*d_6*-DMSO, 400 MHz, 100 MHz)

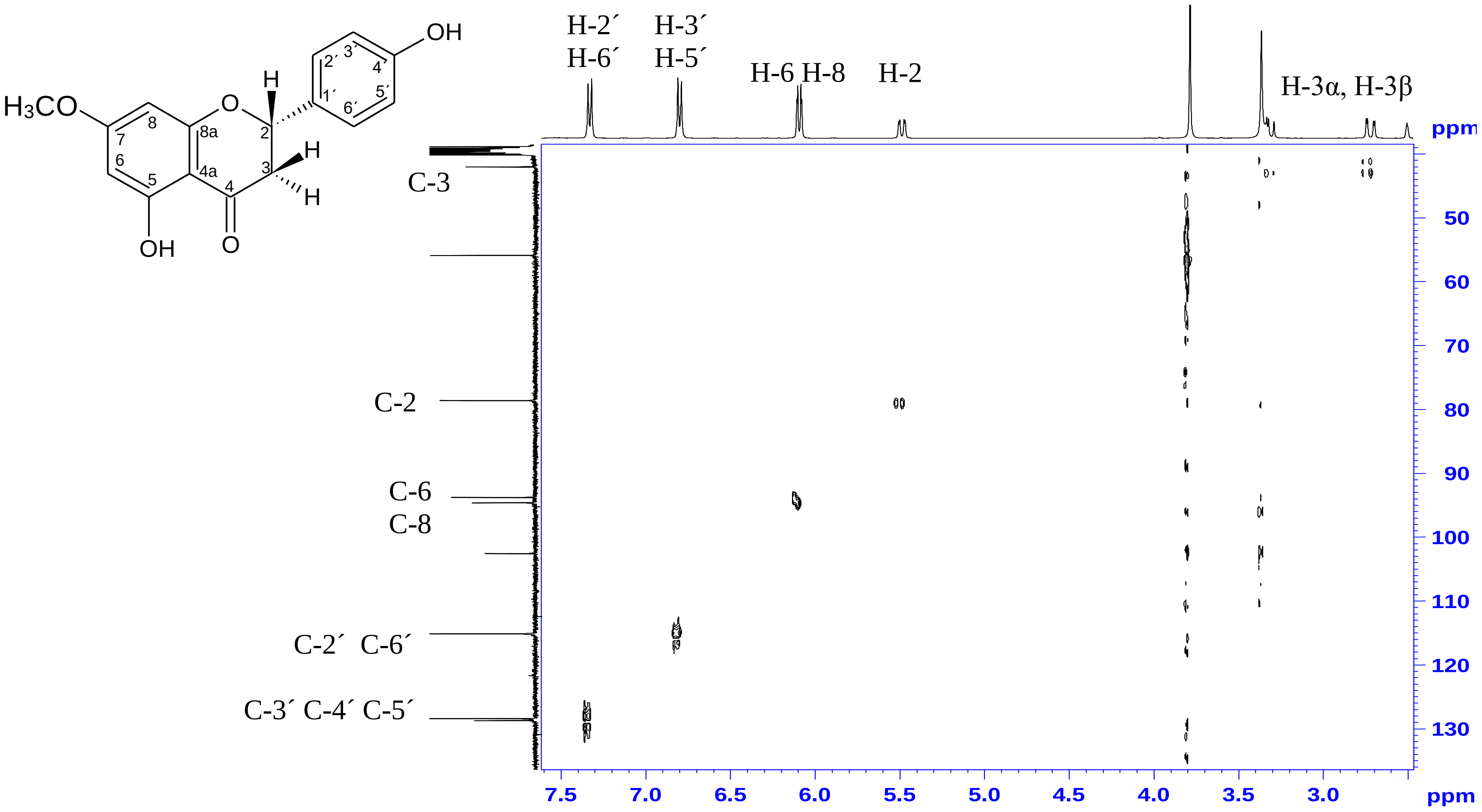

S14: ^{1}H-NMR Spectrum of Cirsimaritin **(3)** d_6-DMSO, 400 MHz

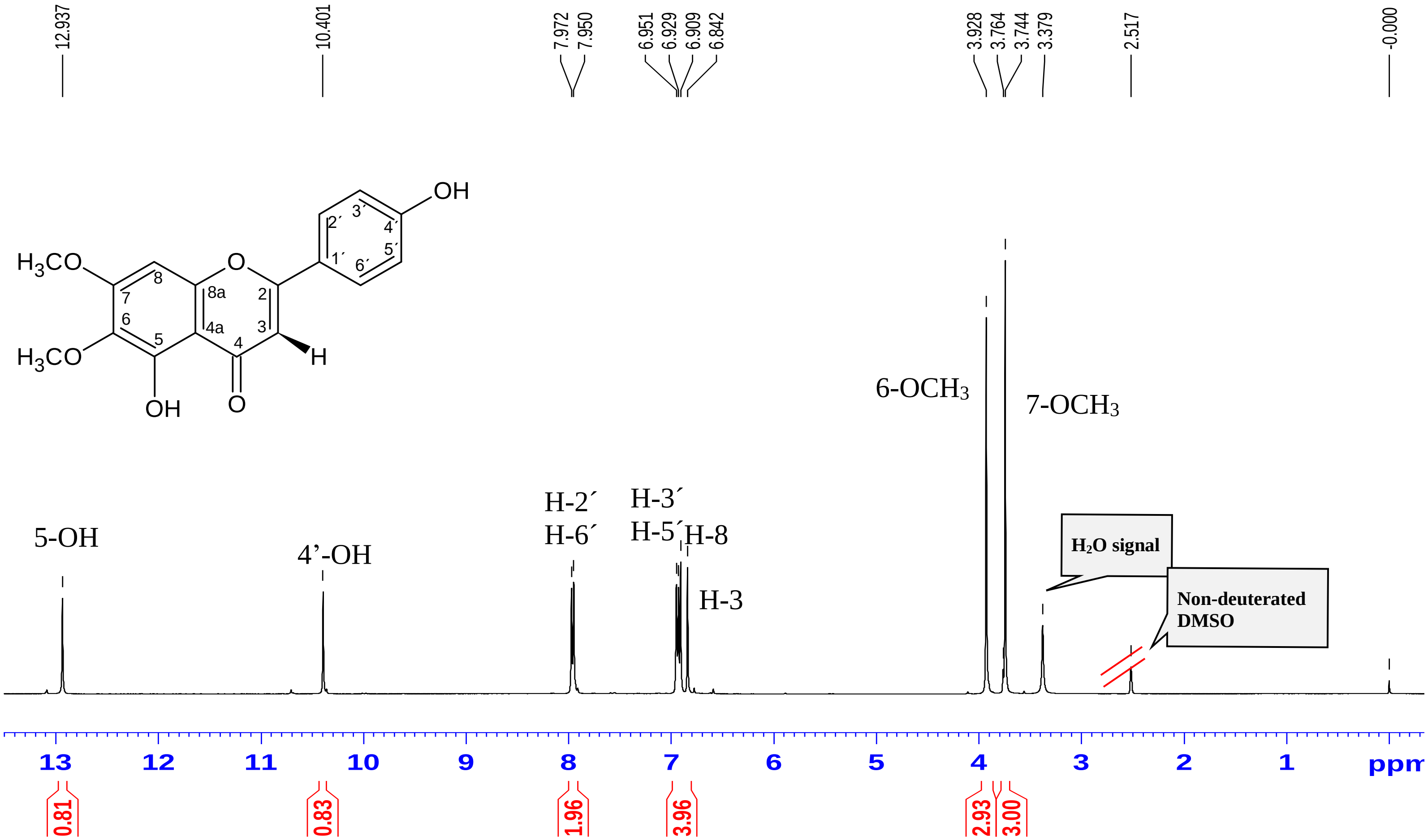

S15: ^{1}H-NMR Spectrum (Ext. 6.8 – 8.0 ppm) of Cirsimaritin (**3**) d_6-DMSO, 400 MHz

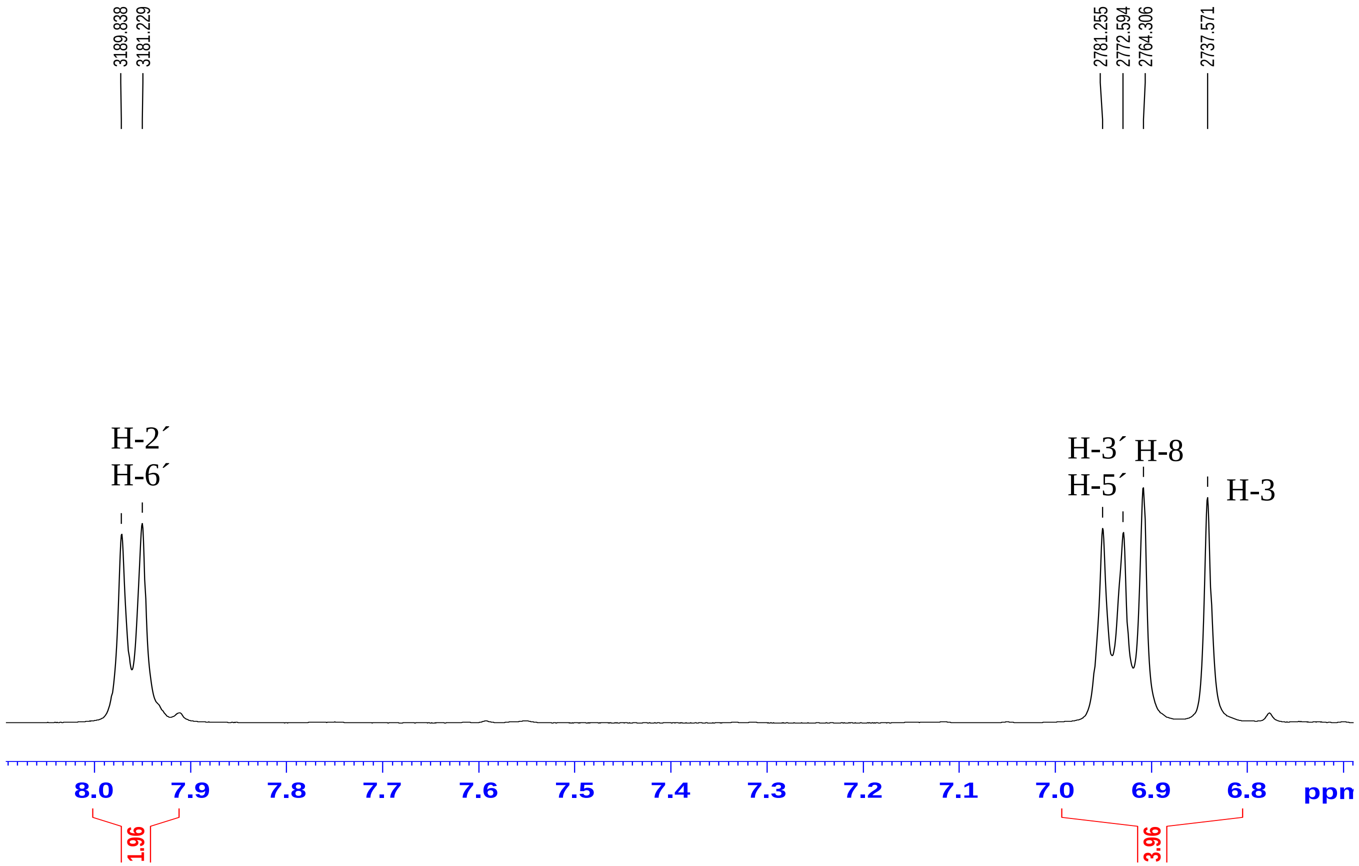

S16: COSY Spectrum of Cirsimaritin (**3**) d_6-DMSO, 400 MHz

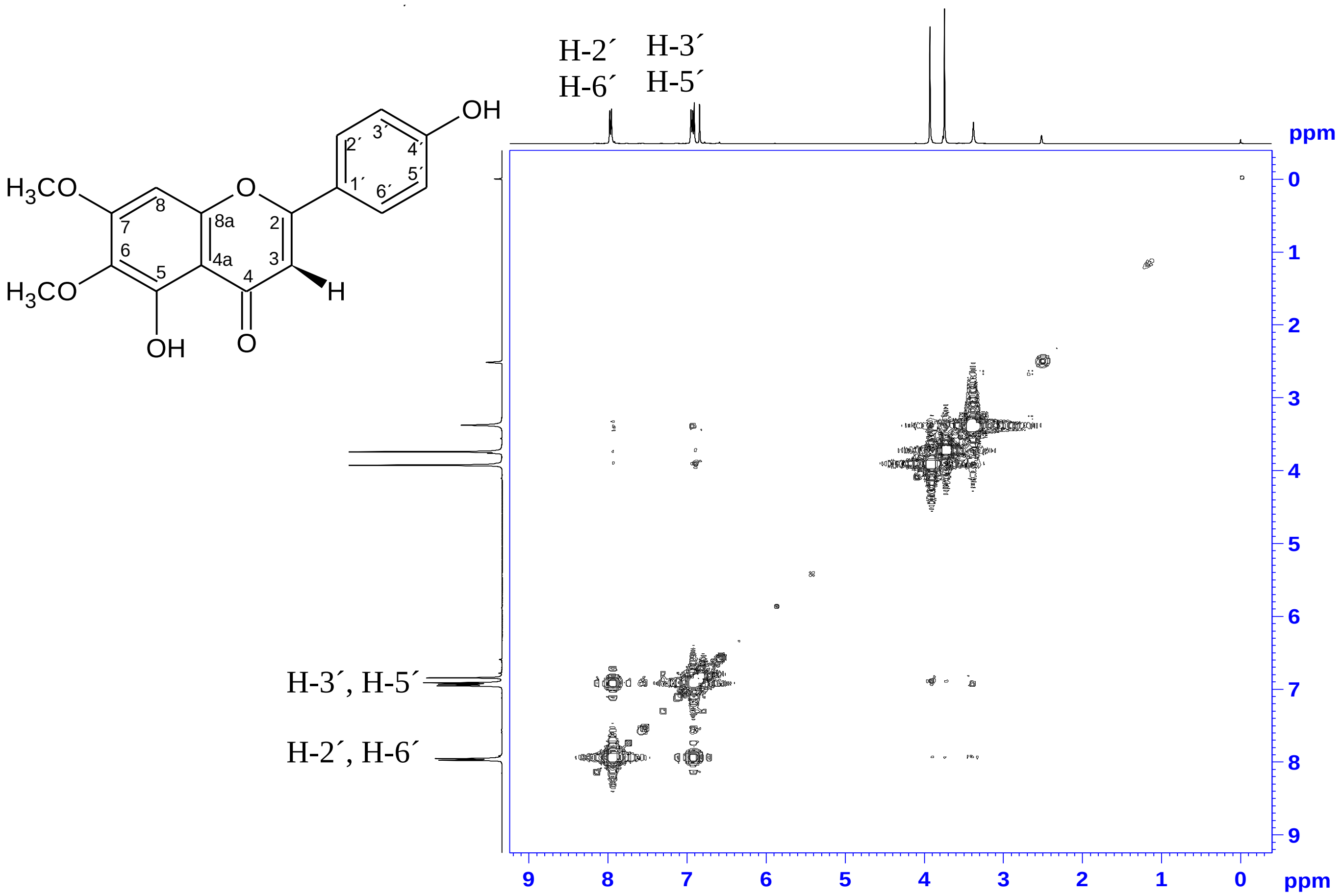

S17: COSY Spectrum (Ext ^{1}H: 5.0-9.4 ppm; ^{1}H: 5.0-9.4 ppm) of Cirsimaritin (**3**) d_6-DMSO, 400 MHz

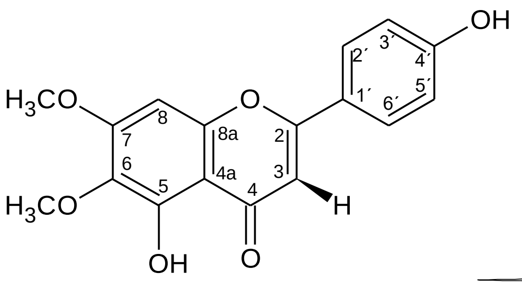

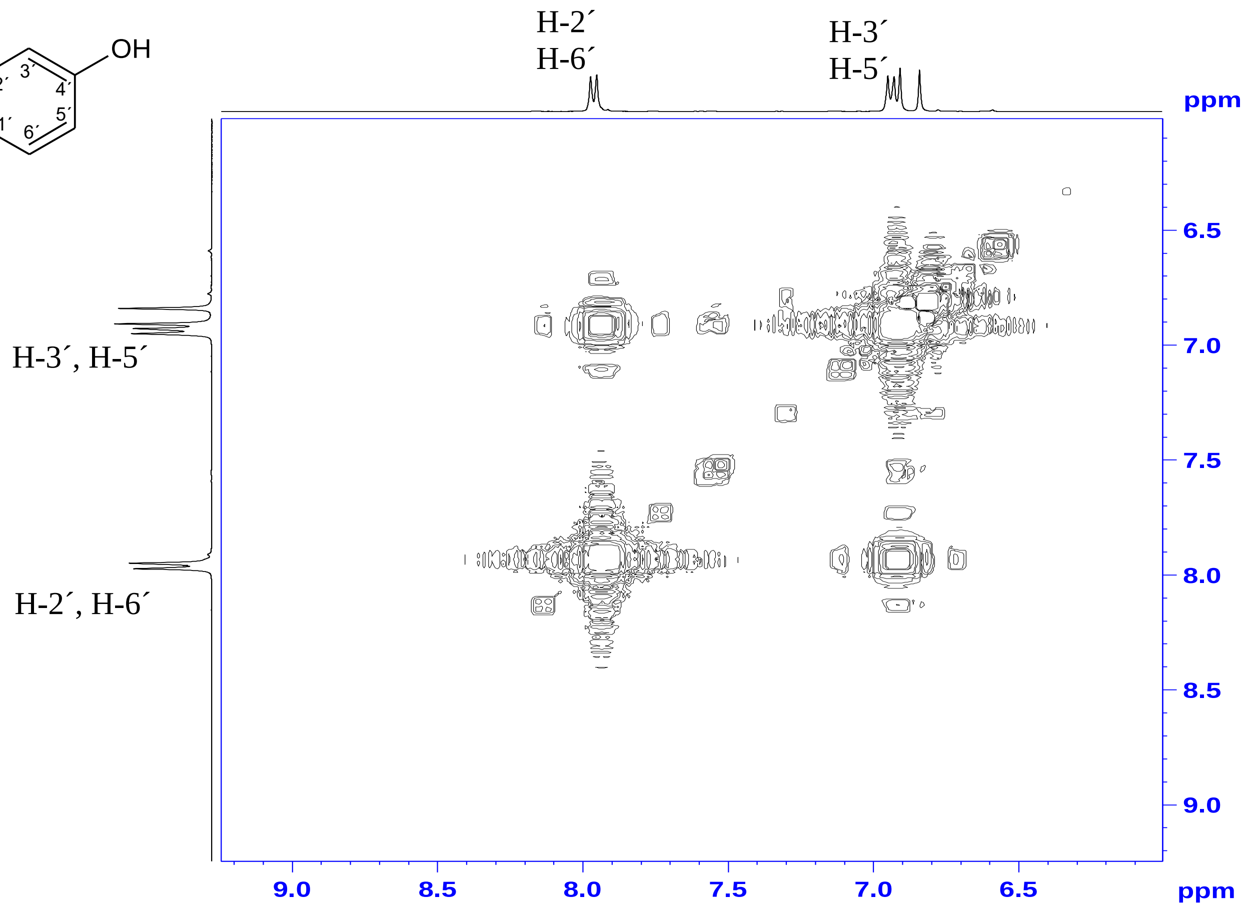

S18: ^{13}C-NMR Spectrum of Cirsimaritin (**3**) (d_6-DMSO, 100 MHz)

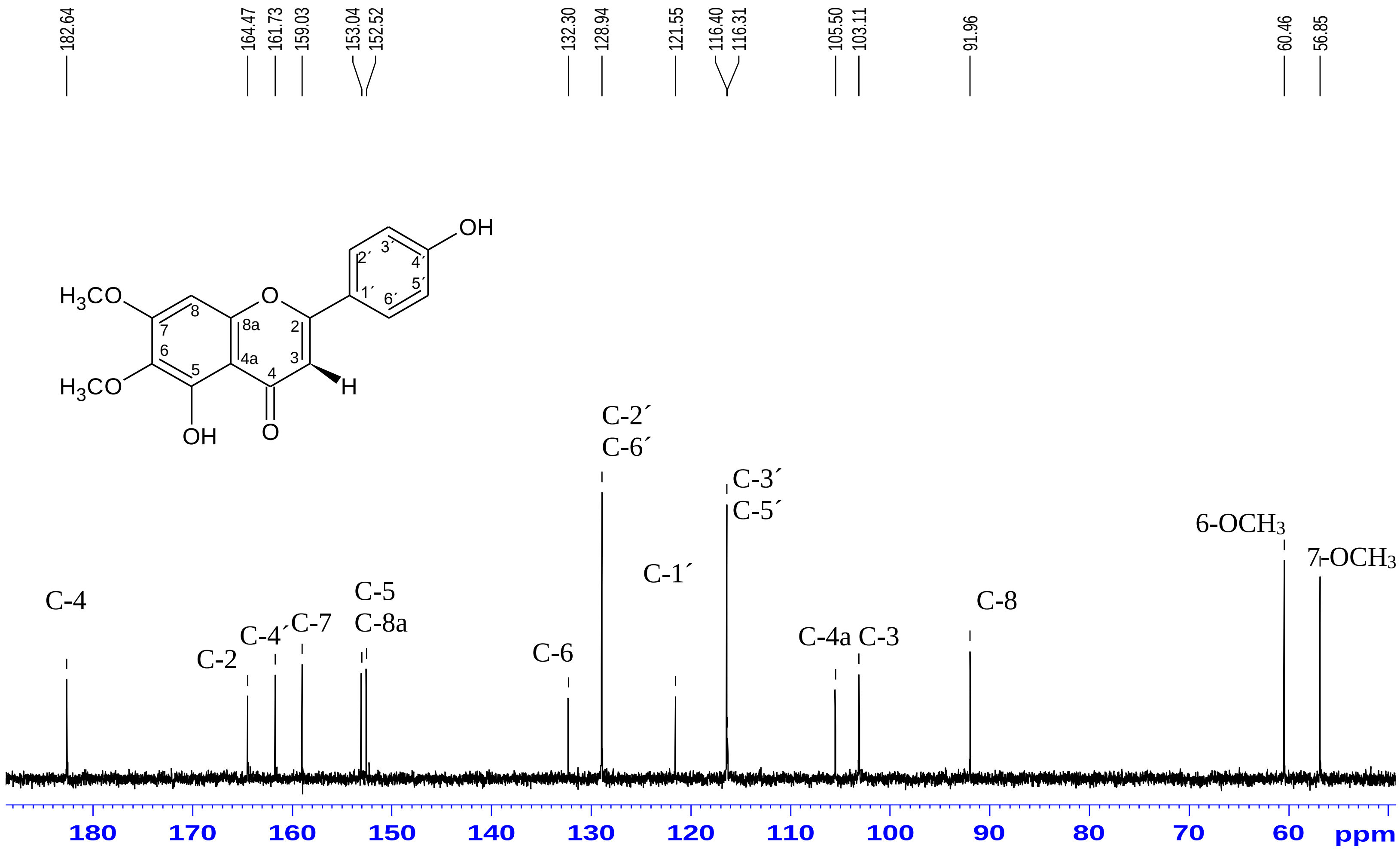

S19: HSQC Spectrum (Ext. ^{1}H: 6.2-8.4 ppm; ^{13}C: 85-145 ppm) of Cirsimaritin (**3**) (d_6-DMSO, 400 MHz, 100 MHz)

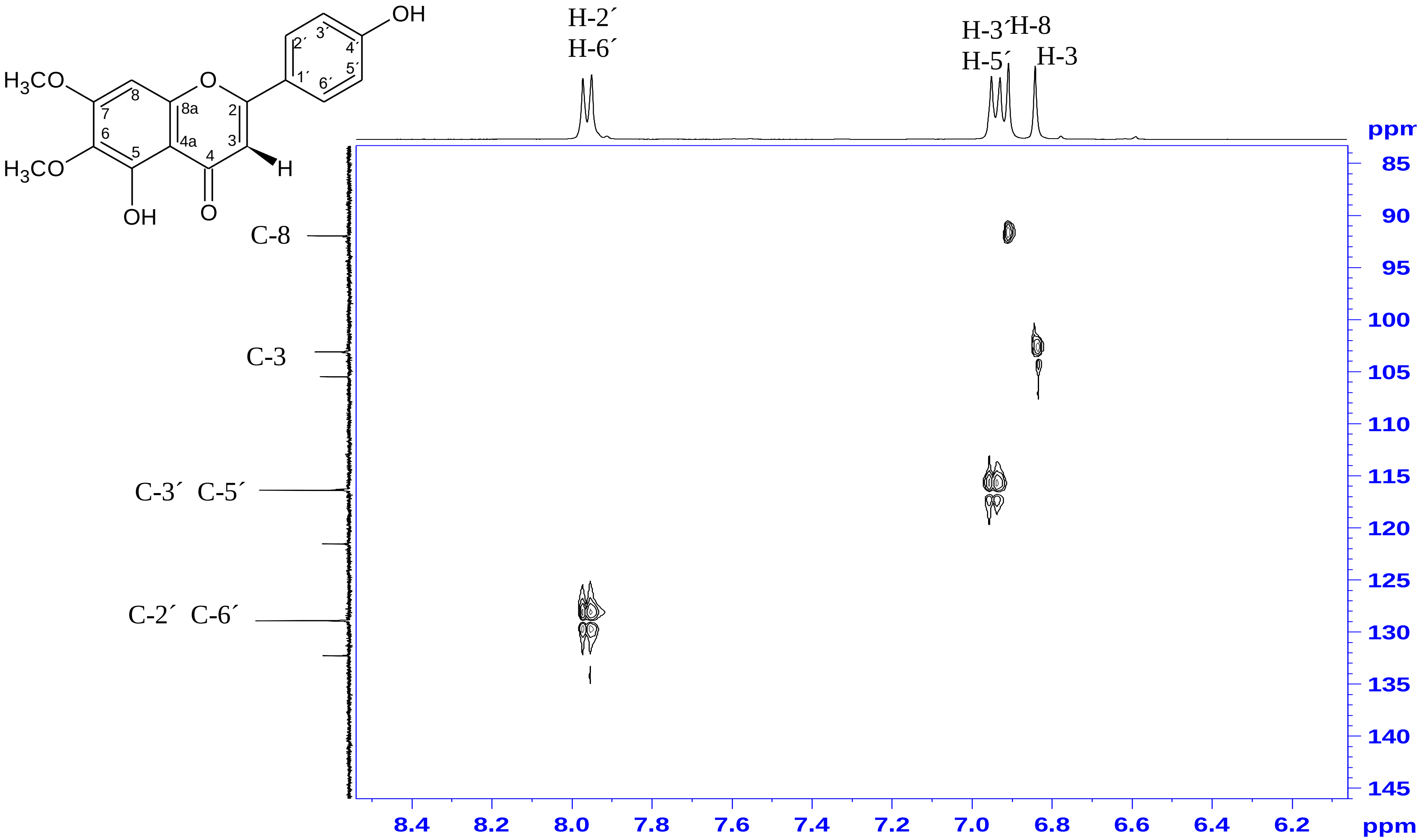

S20: ^{1}H-NMR Spectrum of Naringenin (**4**) d_6-DMSO, 400 MHz

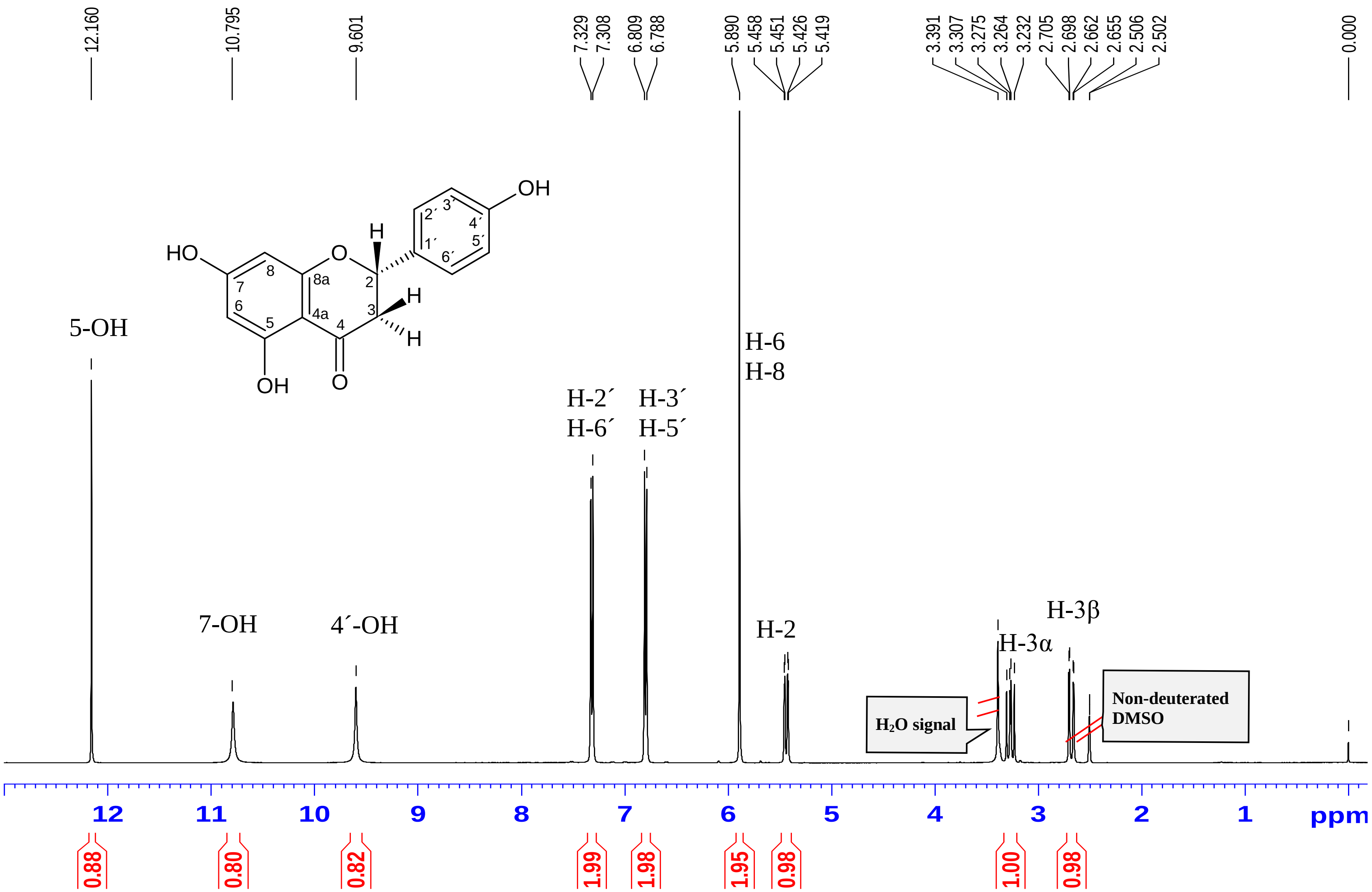

S21: ^{1}H-NMR Spectrum (Ext. 8.0 – 2.0 ppm) of Naringenin (**4**) d_6-DMSO, 400 MHz

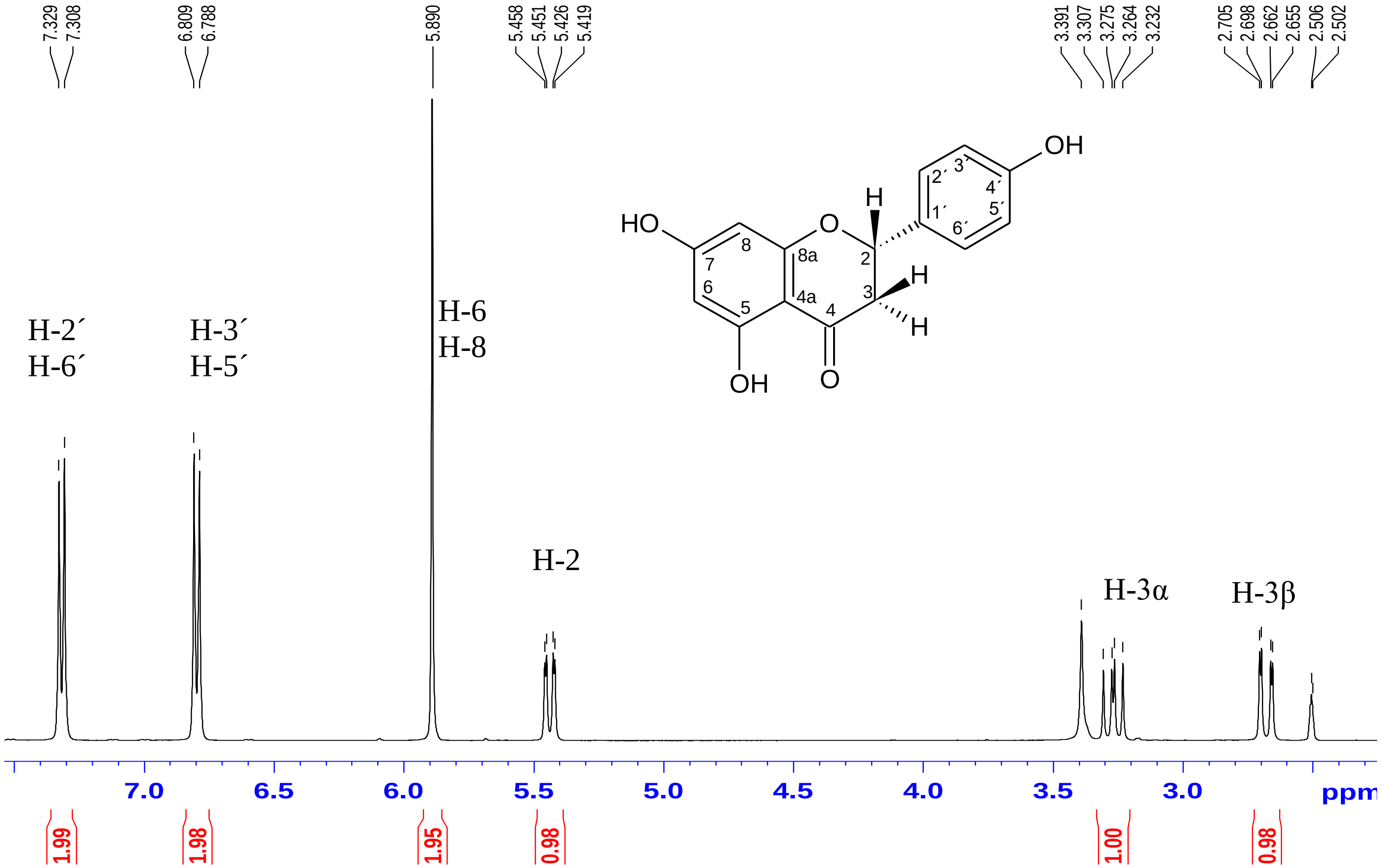

S22: COSY Spectrum of Naringenin (**4**) d_6-DMSO, 400 MHz

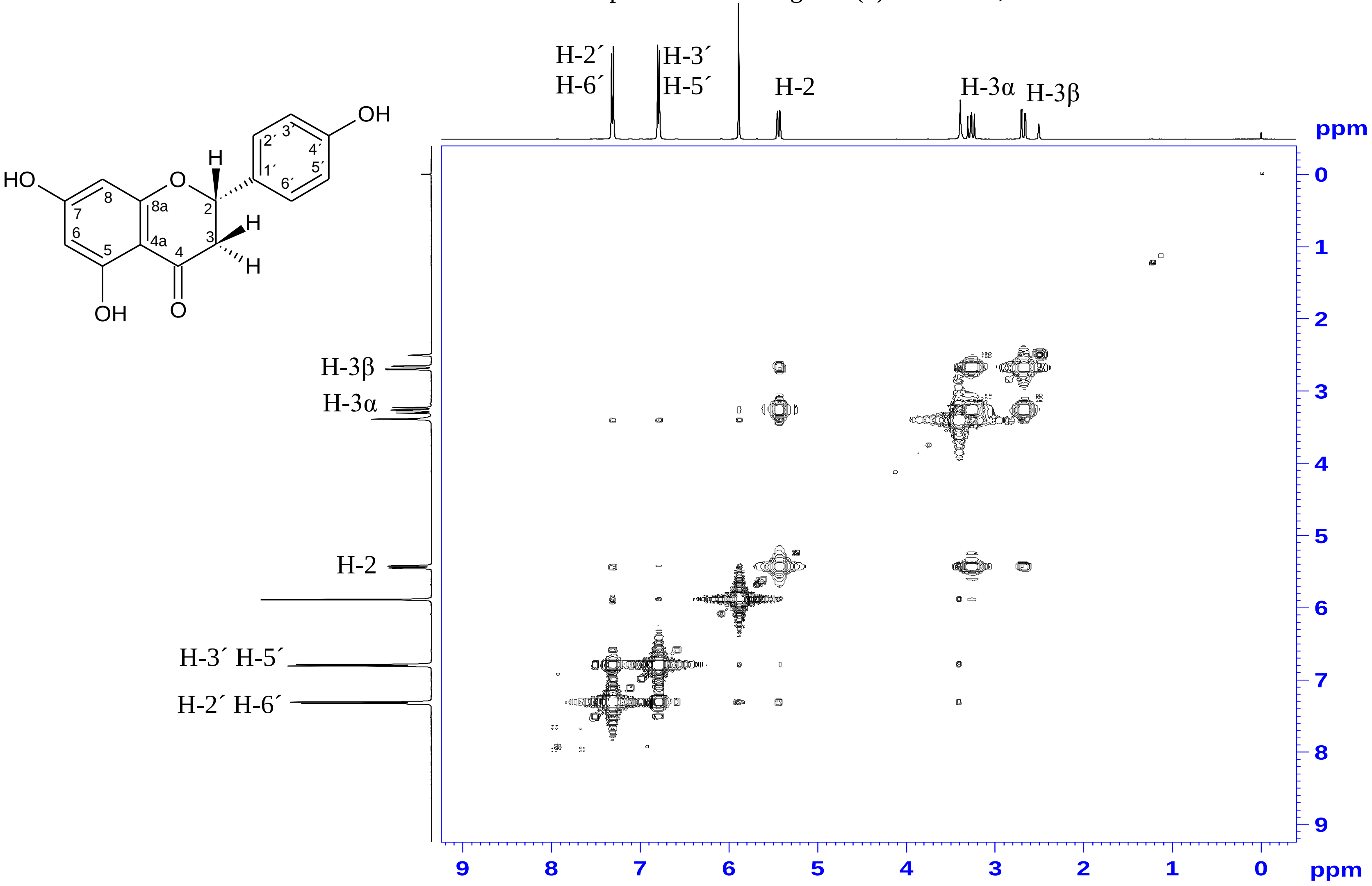

S23: ^{13}C-NMR Spectrum of Naringenin (**4**) (*d*$_6$-DMSO, 100 MHz)

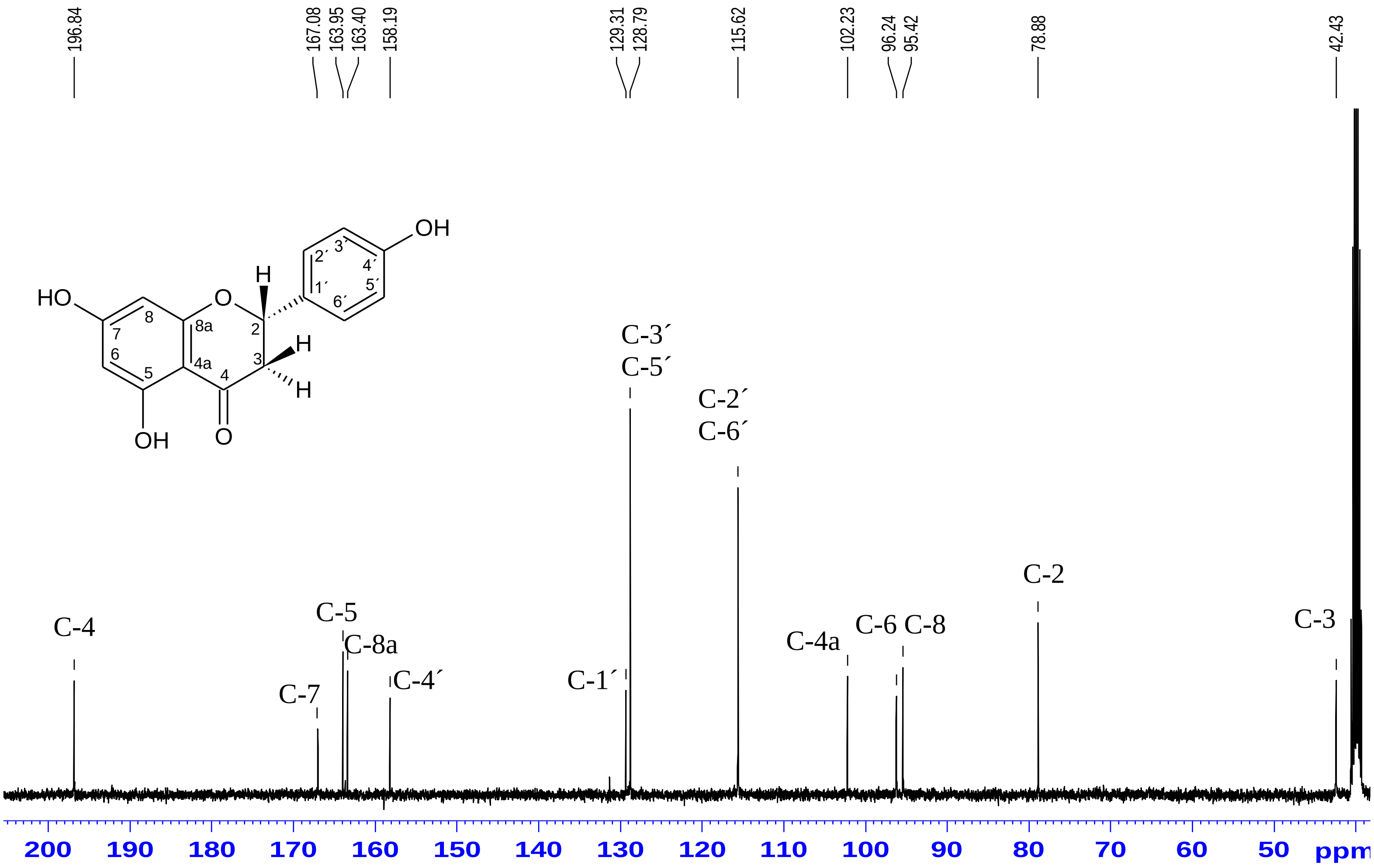

S24: DEPT-135 Spectrum of Naringenin (**4**) (*d*$_6$-DMSO, 100 MHz)

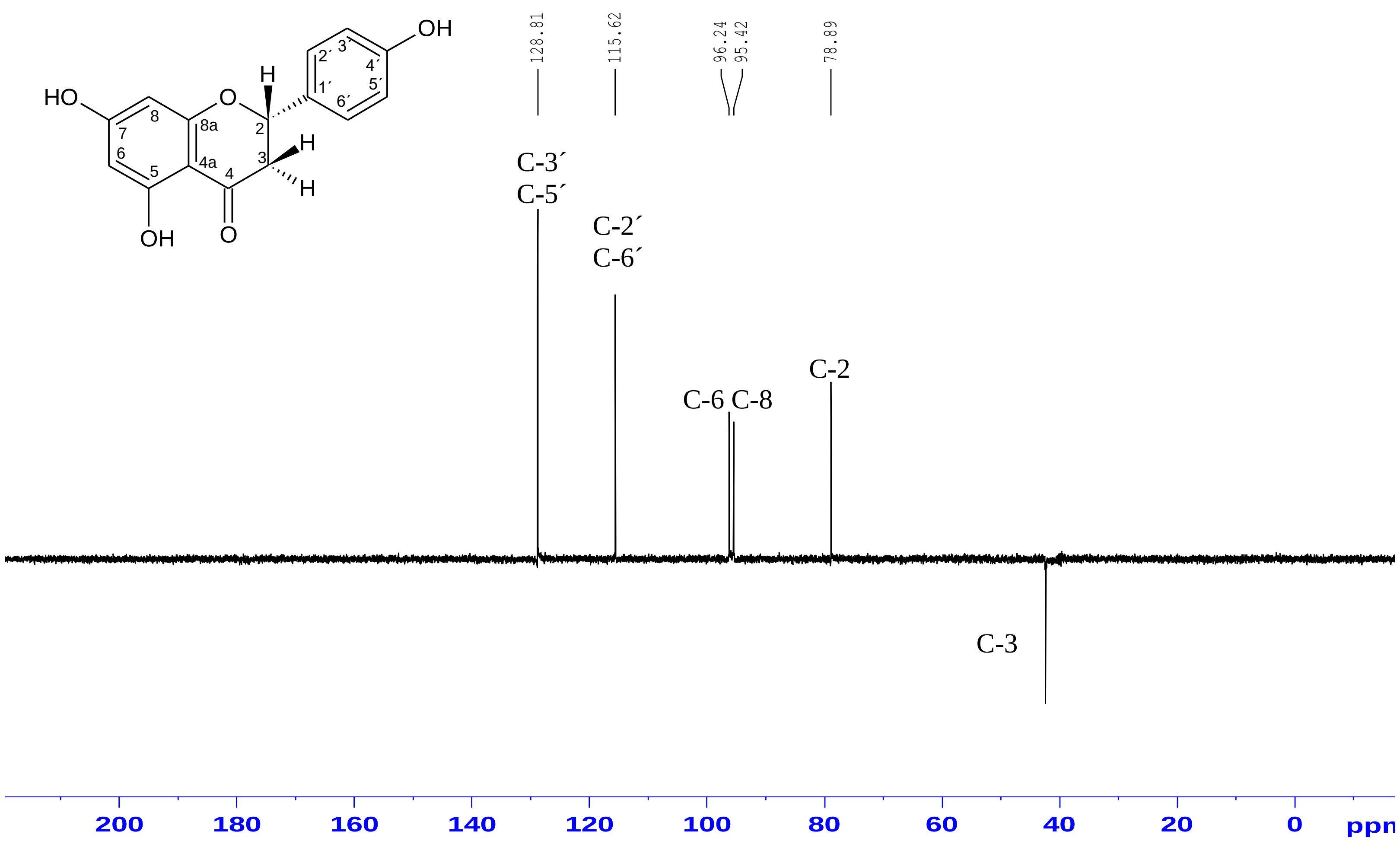

S25: HSQC Spectrum (Ext. ^{1}H: 0-9.0 ppm; ^{13}C: 10-180 ppm) of Naringenin (**4**) (d_6-DMSO, 400 MHz, 100 MHz)

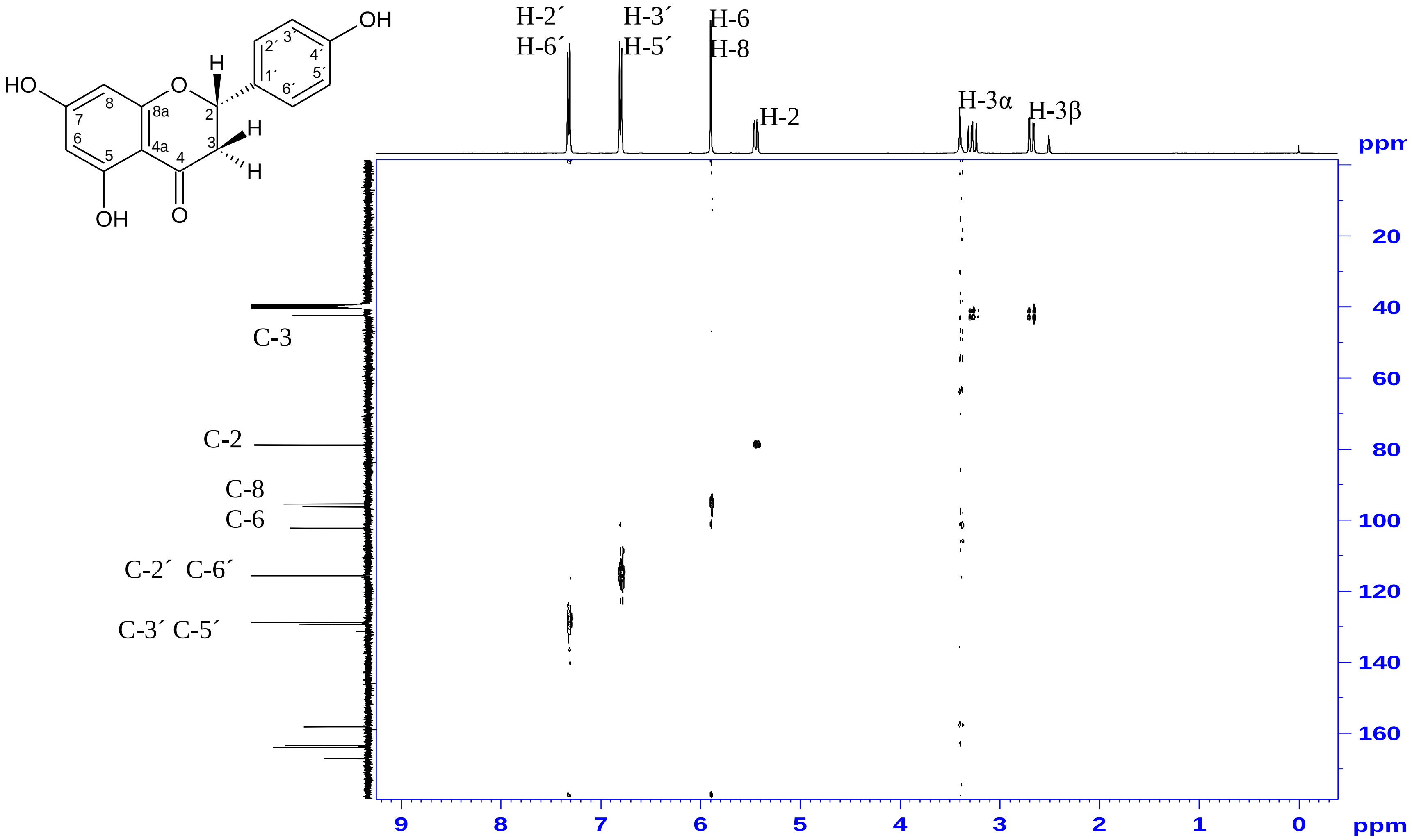

S26: IR spectrum of Pinocembrin **1**

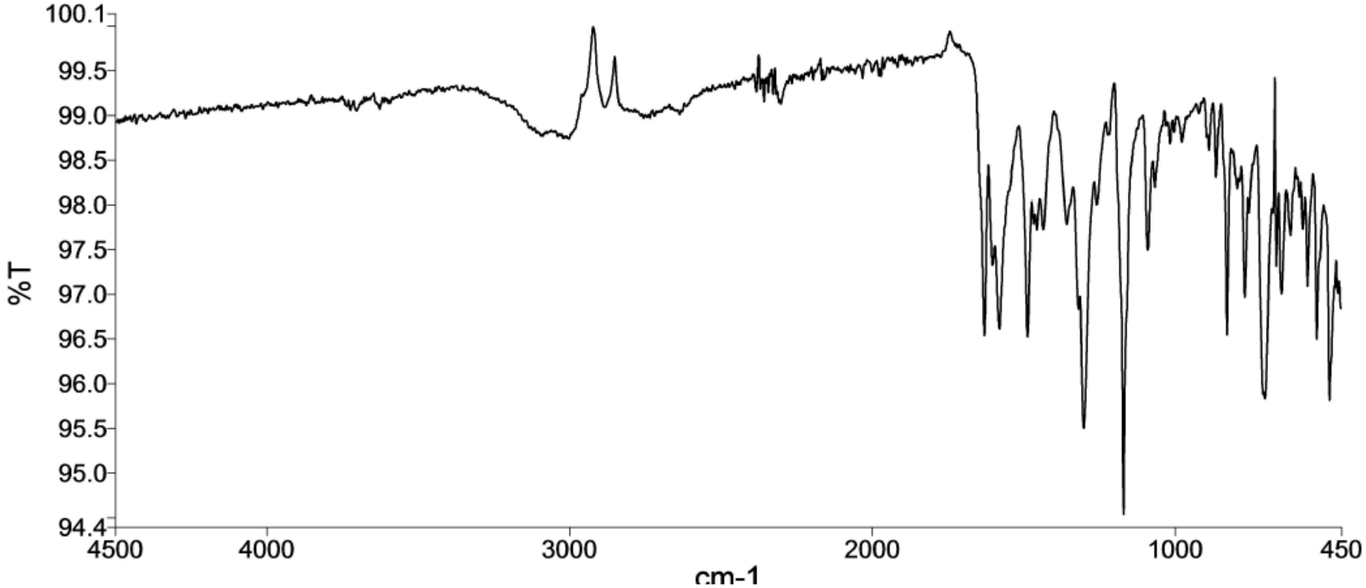

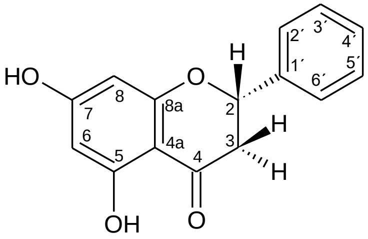

S27: IR spectrum of Sakuranetin **2**

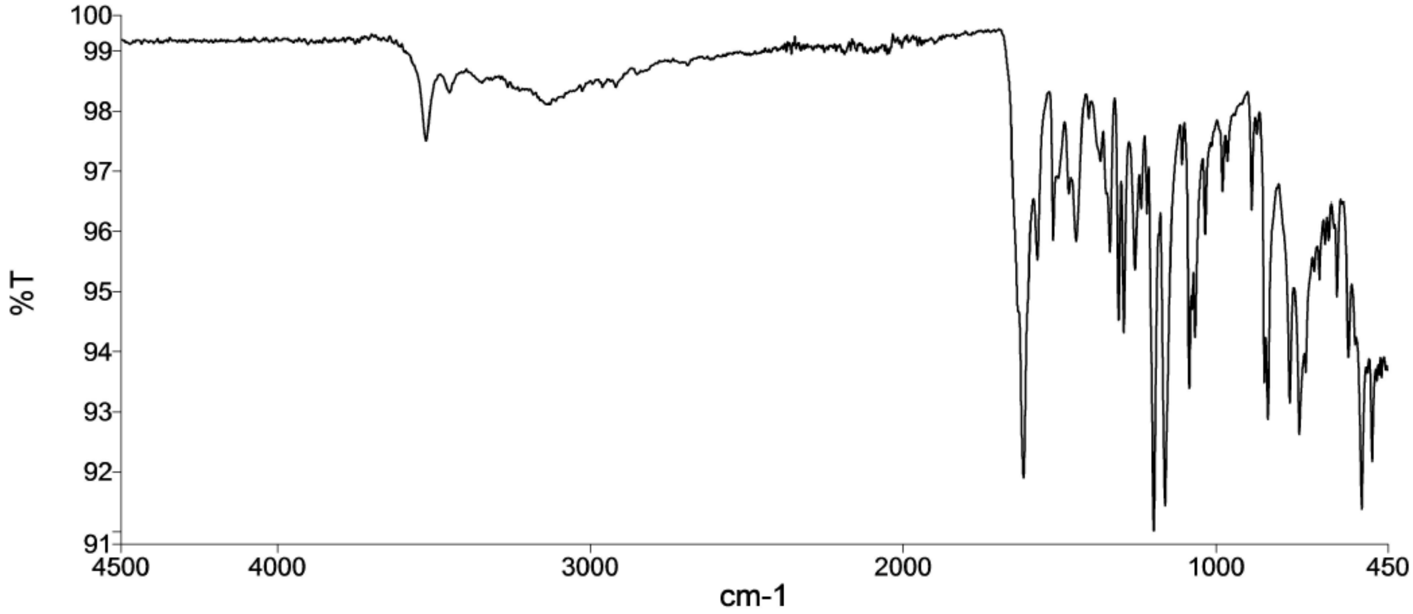

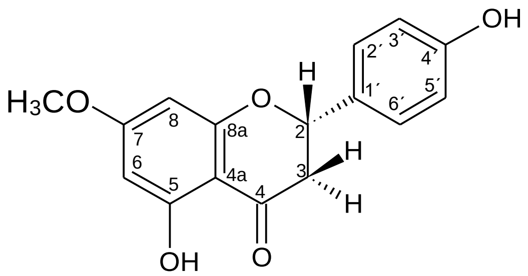

S28: IR spectrum of Cirsimaritin **3**

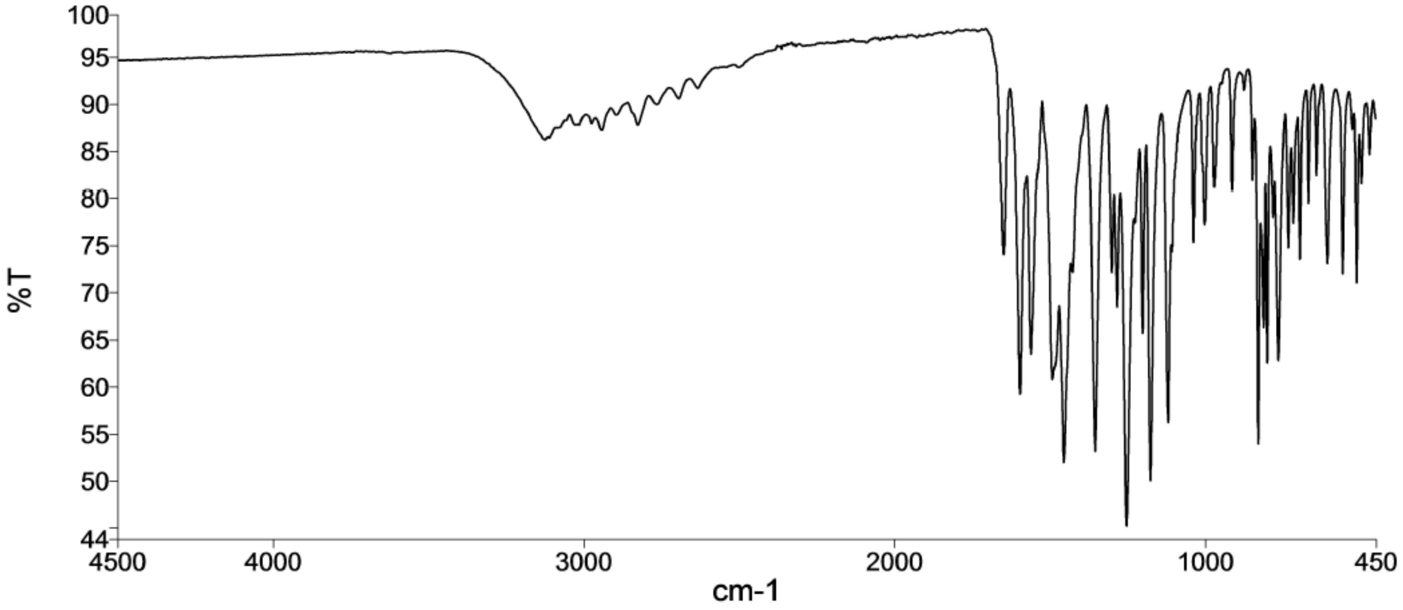

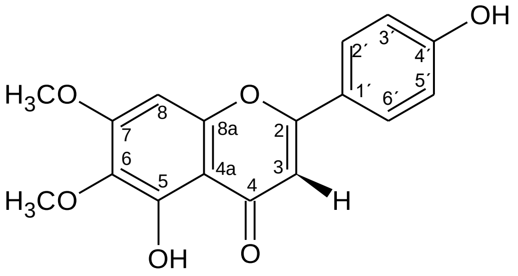

Table 1. ^{1}H-NMR spectral data of compounds 1-4 (400 MHz, DMSO-d_6)

	δ in ppm (m, *J* in Hz)			
**Position**	**1**	**2**	**3**	**4**
**2**	5.59 (dd, 12.71, 2.98)	5.49 (dd, 12.75, 2.86)	-	5.44 (dd, 12.78, 2.88)
**3α(ax)**	3.26 (dd, 17.10, 12.71)	3.33 (dd, 17.15, 12.75)	6.84 (s)	3.27 (dd, 17.11, 12.78)
**3β(ec)**	2.78 (dd, 17.10, 2.98)	2.72 (dd, 17.15, 2.86)	-	2.68 (dd, 17.11 2.88)
**6**	5.94 (d, 1.70)	6.11 (d, 2.09)	-	5.89 (s)
**8**	5.91 (d, 1.70)	6.08 (d, 2.12)	6.91 (s)	5.89 (s)
**2´,6´**	7.52 (d, 7.05)	7.33 (d, 8.48)	7.96 (d, 8.61)	7.32 (d, 8.48)
**3´,5´**	7.45-7.37 (m)	6.80 (d, 8.48)	6.94 (d, 8.61)	6.80 (d, 8.48)
**4´**	7.45-7.37 (m)	-	-	-
**5-OH**	12.14 (s)	12.13 (s)	12.94 (s)	12.16 (s)
**7-OH**	10.88 (br. s)	-	-	10.76 (s)
**4´-OH**	-	9.62 (s)	10.40 (s)	9.60 (s)
**6-OCH_3**	-	-	3.93 (s)	-
**7-OCH_3**	-	3.79 (s)	3.75 (s)	-

Table 2. ^{13}C-NMR spectral data of compounds 1-4 (100 MHz, DMSO-d_6)

	δ in ppm			
**Position**	**1**	**2**	**3**	**4**
**2**	78.38	78.63	164.47	78.88
**3**	42.10	42.03	103.11	42.43
**4**	195.95	196.99	182.64	196.89
**4a**	101.80	102.60	105.50	102.33
**5**	163.50	163.21	153.04	163.95
**6**	95.05	93.79	132.30	95.42
**7**	166.74	167.43	159.03	167.08
**8**	95.94	94.65	91.96	96.24
**8a**	162.72	162.89	152.52	163.40
**1´**	138.70	128.69	121.55	129.31
**2´, 6´**	126.62	128.39	128.94	128.79
**3´,5´**	128.55	115.17	116.40	115.62
**4´**	128.55	157.78	161.73	158.19
**6-OCH_3**			60.46	
**7-OCH_3**		55.89	56.85	
